# Quality of Patient-Centered eHealth Information on Erosive Tooth Wear: Systematic Search and Evaluation of Websites and YouTube Videos

**DOI:** 10.2196/49514

**Published:** 2024-01-31

**Authors:** Lena Holland, Amelie Friederike Kanzow, Annette Wiegand, Philipp Kanzow

**Affiliations:** 1 Department of Preventive Dentistry, Periodontology and Cariology University Medical Center Göttingen Göttingen Germany; 2 Study Deanery University Medical Center Göttingen Göttingen Germany

**Keywords:** consumer health information, dental erosion, dental sciences, digital media, erosive tooth wear, evidence-based dentistry, health education, information quality, internet, shared decision making

## Abstract

**Background:**

Due to the declining prevalence of dental caries, noncarious tooth defects such as erosive tooth wear have gained increased attention over the past decades. While patients more frequently search the internet for health-related information, the quality of patient-centered, web-based health information on erosive tooth wear is currently unknown.

**Objective:**

This study aimed to assess the quality of patient-centered, web-based health information (websites and YouTube videos) on erosive tooth wear.

**Methods:**

German-language websites were systematically identified through 3 electronic search engines (google.de, bing.de or yahoo.de, and duckduckgo.com) in September 2021. Eligible websites were independently assessed for (1) technical and functional aspects via the LIDA instrument, (2) readability via the Flesch reading-ease score, (3) comprehensiveness of information via a structured checklist, and (4) generic quality and risk of bias via the DISCERN instrument by 2 different reviewers. An overall quality score (ie, higher scores being favored) generated from all 4 domains was used as the primary outcome. Quality scores from each domain were separately analyzed as secondary outcomes and compared by the Friedman test. The effect of practice-specific variables on quality scores of websites from private dental offices was assessed using generalized linear modeling. Eligible YouTube videos were judged based on (1) the comprehensiveness of information, (2) viewers’ interaction, and (3) viewing rate. The comprehensiveness of information was compared between websites and YouTube videos using the Wilcoxon rank-sum test.

**Results:**

Overall, 231 eligible websites and 7 YouTube videos were identified and assessed. The median overall quality of the websites was 33.6% (IQR 29.8%-39.2%). Secondary outcome scores amounted to 64.3% (IQR 59.8%-69.0%) for technical and functional aspects, 40.0% (IQR 34.0%-49.0%) for readability, 11.5% (IQR 3.9%-26.9%) for comprehensiveness of information, and 16.7% (IQR 8.3%-23.3%) for generic quality. While the comprehensiveness of information and generic quality received low scores, technical and functional aspects as well as readability resulted in higher scores (both *P*_adjusted_<.001). Regarding practice-specific variables, websites from private dental offices outside Germany (*P*=.04; B=–6.64, 95% CI –12.85 to –0.42) or from dentists who are a dental society member (*P*=.049; B=–3.55, 95% CI –7.09 to –0.01) resulted in lower readability scores (ie, were more difficult to read), while a shorter time since dentists’ examination resulted in higher readability scores (*P*=.01; B=0.24 per year, 95% CI 0.05-0.43). The comprehensiveness of information from YouTube videos was 34.6% (IQR 13.5%-38.5%). However, the comprehensiveness of information did not vary between websites and YouTube videos (*P*=.09). Additionally, viewers’ interaction (1.7%, IQR 0.7%-3.4%) and viewing rates (101%, IQR 54.6%-112.6%) were low.

**Conclusions:**

The quality of German-language, patient-centered, web-based information on erosive tooth wear was limited. Especially, the comprehensiveness and trustworthiness of the available information were insufficient. Web-based information on erosive tooth wear requires improvement to inform patients comprehensively and reliably.

## Introduction

Patients search the internet for health-related information to an increased extent [1], and web-based health information impact the physician-patient relationship [2]. High-quality content empowers patients to participate in an informed shared decision-making process [3]. To be beneficial for patients, web-based health information should be easily accessible and readable, comprehensive, and trustworthy (ie, being credible and unbiased).

In addition, dental patients often search for dental or oral conditions and treatment procedures using the internet [4,5]. Within the context of preventive and restorative dentistry, multiple studies focused on patient-centered, web-based health information. Each study assessed information on specific oral conditions and treatment procedures, including endodontics [6-11], dental caries [12-17], pit and fissure sealant application [18], restoration repair [19], and periodontitis [20-24].

Due to the declining prevalence of dental caries over the past decades [25], noncarious tooth defects such as erosive tooth wear have gained increased attention. Erosive tooth wear is defined as tooth wear with dental erosion as the primary etiological factor. While tooth decay is caused by acids derived from oral microorganisms, dental erosion is the loss of mineralized tooth substance due to exposure to acids not derived from oral bacteria [26]. The erosive demineralization might be caused either by extrinsic (eg, acids in food or beverages) or intrinsic (ie, endogenous acid) acids [27]. A recent review estimated a global prevalence between 20% and 45% for permanent teeth [28]. In Germany, a prevalence of 44.8% in adults aged 35 to 44 years was reported [29]. Advanced erosive tooth wear often requires restorative management by direct or indirect dental restorations to reduce pain and dentine hypersensitivity and to restore esthetic and function [30].

To the best of our knowledge, no previous study assessed patient-centered, web-based information on noncarious tooth defects such as erosive tooth wear. Consequently, it is currently unknown whether patients might find comprehensive and trustworthy information on erosive tooth wear using the internet.

Therefore, this study aimed to analyze patient-centered, web-based health information (ie, websites and YouTube videos) on erosive tooth wear published by different content providers and to assess its quality and credibility. Websites were scored across four domains: (1) technical and functional aspects, (2) readability, (3) comprehensiveness of information, and (4) generic quality and risk of bias. YouTube videos were judged based on (1) comprehensiveness of information, (2) viewers’ interaction, and (3) viewing rate. Another objective was to identify practice-specific variables, which might impact the quality of websites provided by private dental offices. The null hypotheses were that information quality and credibility do not vary across different content providers, and practice-specific variables of private dental offices do not impact information quality and credibility.

## Methods

### Ethical Considerations

The reporting of this study is in accordance with the PRISMA (Preferred Reporting Items for Systematic Reviews and Meta-Analyses) and the ENTREQ (Enhancing Transparency in Reporting the Synthesis of Qualitative Research) statements [31,32]. The study was approved by the local ethics committee of the University Medical Center Göttingen (approval 3/11/23). Only publicly available, nonsensitive data were used.

### Eligible Sources

Freely accessible websites and YouTube videos in German language published from (1) private dental offices; (2) corporate dental offices or private hospital groups resembling juridical entities (ie, Medizinisches Versorgungszentrum, Gesellschaft mit beschränkter Haftung, and Aktiengesellschaft); (3) public dental clinics or dental schools; (4) dental societies, dental regulatory bodies, public bodies, or insurance companies; or (5) information services (ie, other commercial or nonprofit content providers) containing patient-centered health information on erosive tooth wear were regarded as eligible sources.

Websites and YouTube videos from other content providers (eg, dental laboratories, dental supply and material companies, and manufacturers of dental hygiene products), advertisements, articles, books, research agencies without focus on patient care, and forums or blogs operated by nondentists were excluded. In addition, YouTube videos without sound or captions and videos with a duration of >15 minutes were not considered as eligible sources.

### Search Strategy

Websites were identified through 3 electronic search engines (google.de, bing.de or yahoo.de, and duckduckgo.com). The searches were performed on September 21 and 22, 2021, using 6 different search terms representing different German synonyms for male and female dentists, dentists, dental offices, dental erosion, erosive tooth wear, and acid-related tooth destruction, in both professional and lay language (Table 1). The same search terms were used to identify YouTube videos from Youtube.de on September 29, 2021.

**Table 1:** Search terms used and number of results for each search engine.

Search term^a^	Google (google.de), n	Bing or Yahoo (bing.de or yahoo.de), n	DuckDuckGo (duckduckgo.com), n
(Zahnärztin OR Zahnarzt OR Zahnärzte OR Zahnarztpraxis) Erosion	200	602	206
(Zahnärztin OR Zahnarzt OR Zahnärzte OR Zahnarztpraxis) Erosionen	200	470	224
(Zahnärztin OR Zahnarzt OR Zahnärzte OR Zahnarztpraxis) “erosive Zahnhartsubstanzdefekte”	34	27	30
(Zahnärztin OR Zahnarzt OR Zahnärzte OR Zahnarztpraxis) “erosiver Zahnhartsubstanzverlust”	25	18	15
(Zahnärztin OR Zahnarzt OR Zahnärzte OR Zahnarztpraxis) “Säureschädigung”	41	62	40
(Zahnärztin OR Zahnarzt OR Zahnärzte OR Zahnarztpraxis) “Säureschäden”	157	283	177

^a^Search terms represent different German synonyms for male and female dentists, dentists, dental offices, dental erosion, erosive tooth wear, and acid-related tooth destruction, in both professional and lay language.

For all searches, a computer running Windows 10 Home (Microsoft Inc) and Firefox version 92.0 (Mozilla Foundation) connected to the internet in Germany was used. Prior to each search, the browser cache, cookies, and browser history were cleared. All searches were performed using search engines’ standard settings. Only web pages being displayed as “most relevant” were considered.

Based on the search results and shown previews, web pages not meeting the inclusion criteria were excluded, and potentially eligible web pages were screened in full. Subsequently, noneligible web pages and duplicates were removed. Multiple web pages published under the same domain were jointly assessed as 1 website. The search and inclusion of websites was performed by 1 author (LH) and verified by another author (PK). Any uncertainties were resolved through discussion.

### Data Extraction

The following data were extracted from each website or YouTube video using a pilot-tested spreadsheet (Multimedia Appendix 1), if available: (1) content provider’s name, (2) URL, (3) content provider’s country (ie, Germany or foreign country), (4) content provider’s location (ie, rural, town [<100,000 inhabitants], or city [≥100,000 inhabitants]), (5) content provider (ie, private dental office, corporate dental office or private hospital group, public dental clinic or dental school, dental society or dental regulatory body or public body or insurance company, or information service).

If a website or YouTube video was published from a private dental office, the following data were also extracted: (1) practice setting (ie, single practitioner or multiple dentists), (2) sex (ie, female, male, or mixed [in case of multiple dentists]), (3) dental society memberships, and (4) years of examination (averaged in case of multiple dentists). In addition, in the case of YouTube videos, (1) upload date, (2) duration (minutes), (3) number of likes, (4) number of dislikes, and (5) number of comments were extracted. Regarding dental society memberships, no differentiation between different fields (eg, restorative dentistry) was made.

If information on memberships in dental associations was missing or unclear, it was compared with the publicly available membership data of the German Society of Dentistry and Oral Medicine (*Deutsche Gesellschaft für Zahn-, Mund- und Kieferheilkunde*; DGZMK) [33] and the Swiss Dental Association (SSO) [34]. In addition, the years of examination were cross-referenced from older versions of the respective websites accessed via the Internet Archive’s Wayback Machine [35], the information provided from dental regulatory bodies, or publicly available curriculum vitae (ie, published in dentists’ dissertations, social networking sites XING or LinkedIn, profiles at doktor.ch [36], or directories of professional representations). Data extraction was independently performed by 2 authors (LH and PK). Subsequently, any discrepancies were resolved by repeated data extraction.

### Outcomes

Quality and credibility of health information on websites were assessed across four domains: (1) technical and functional aspects, (2) readability, (3) comprehensiveness of information, and (4) generic quality and risk of bias. Domains 1, 2, and 4 were scored using established and validated tools. Domain 1 was scored using the LIDA validation instrument (version 1.2; Minervation) for judging the accessibility (1.1), usability (1.2), and reliability (1.3) of health care websites [37]. Domain 2 was scored according to the following equation:

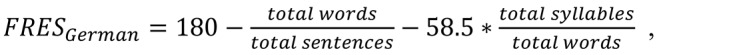



which represents the Flesch reading-ease score (FRES) [38], adapted by Amstad [39] for the German language. Domain 4 was scored using the DISCERN instrument for judging the reliability (4.1) and quality (4.2) of written consumer health information [40].

For domain 3, a structured checklist with 4 subdomains focusing on the etiology and pathogenesis (3.1), clinical signs (3.2), preventive measures (3.3), and therapy options (3.4) of erosive tooth wear was developed by 2 expert clinicians (AW and PK).

The manually scored domains (ie, domains 1, 3, and 4) were independently evaluated by multiple authors (domains 1, 3, and 4: LH; domains 1 and 4: AFK; and domain 3: PK) by applying the scoring guidelines and definitions of each tool. For each tool, the originally published scales and scoring criteria were used. Finally, any discrepancies were resolved through discussion. Domain 2 was evaluated using an automated free web-based tool [41] calculating the FRES on a scale ranging from 0 to 100.

For YouTube videos, the comprehensiveness of information was also explored. Again, 2 reviewers (LH and PK) independently assessed each video based on the same structured checklist. Furthermore, viewers’ interaction and the viewing rate were calculated according to the following equations:

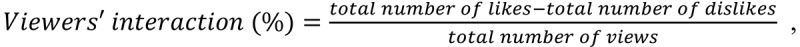

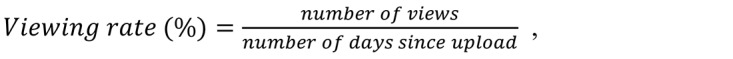





which have been described previously [42].

### Statistical Analysis

Statistical analysis was performed using SPSS Statistics (Macintosh version 29.0.0.0; IBM Corp). The level of statistical significance was set at *P*<.05.

For all manually scored domains (ie, domains 1, 3, and 4), interrater reliability was analyzed using 2-way agreement, average intraclass correlation (ICC[A,2]) [43]. As the scales and numbers of items differed between each domain, relative percentage scores of the maximum possible score sums were used to allow for comparison between domains. While an overall quality score for each website was calculated by averaging the results from all 4 domains (primary outcome), relative percentage scores from each domain were used as secondary outcomes.

Descriptive analysis of quality scores consisted of median, IQR, and range between minimum and maximum scores. Potential differences between each quality domain were assessed using the Friedman test followed by the Dunn-Bonferroni post hoc tests. In addition, statistical differences across content providers (ie, private dental offices, corporate dental offices or private hospital groups, public dental clinics or dental schools, dental societies or dental regulatory bodies or public bodies or insurance companies, or information services) were analyzed by Kruskal-Wallis rank-sum tests followed by Dunn-Bonferroni post hoc tests. For websites posted by private dental offices, the impact of practice-specific variables on both the primary and secondary outcomes was assessed using generalized linear modeling (GLM). Within the multivariable analysis, covariates were entered simultaneously, and only main effects without interaction terms were tested. In case of missing information regarding dental society memberships and years of examination for all dentists, the dental offices’ respective variables were either scored as “no” or websites were excluded from the multivariable analysis (n=9). Finally, the comprehensiveness of information was compared between websites and YouTube videos using the Wilcoxon rank-sum test.

## Results

### Overview

**Figure 1:**
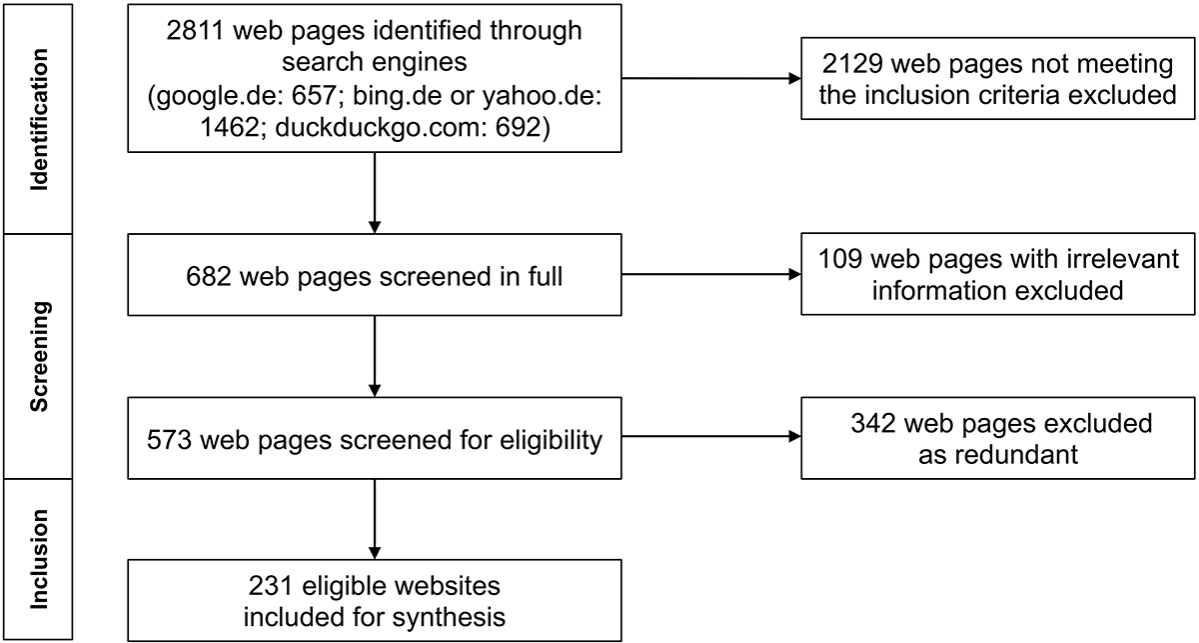
Flowchart representing the systematic workflow of the evaluation.

A total of 231 eligible websites and 7 YouTube videos were identified. A flowchart representing the search is shown in Figure 1.

### Demographic Data

Most websites and YouTube videos were operated by private dental offices (181 websites and 3 YouTube videos). Characteristics of included websites and YouTube videos are shown in Table 2.

**Table 2:** Characteristics of included websites and YouTube videos.

Variables and attributes		Websites (n=231), n (%)	YouTube videos (n=7), n (%)
**Country**			
	Germany	202 (87.4)	5 (71.4)
	Foreign	29 (12.6)	2 (28.6)
**Content provider**			
	Private dental office	181 (78.4)	3 (42.9)
	Corporate dental office or private hospital group	20 (8.7)	2 (28.6)
	Public dental clinic or dental school	6 (2.6)	0 (0)
	Dental society, dental regulatory body, public body, or insurance company	16 (6.9)	0 (0)
	Information service	8 (3.5)	2 (28.6)

Among the websites (n=181) published by private dental offices, most dental offices were located in cities with ≥100,000 inhabitants (n=76, 42%), and multiple dentists worked together (n=109, 60.2%) in a mixed sex setting (n=80, 44.2%). In 61.9% (n=112) of the private dental offices, at least 1 dentist was a member of a dental society. Further details on dentists’ demographics are shown in Table 3.

**Table 3:** Dentists’ demographics of included websites and YouTube videos operated by private dental offices.

Variable and attributes			Websites (n=181)		YouTube videos (n=3)
**Country, n (%)**					
	Germany	166 (91.7)		3 (100)	
	Foreign	15 (8.3)		0 (0)	
**Practice location, n (%)**					
	Rural	35 (19.3)		1 (33)	
	Town (<100,000 inhabitants)	70 (38.7)		2 (67)	
	City (≥100,000 inhabitants)	76 (42)		0 (0)	
**Practice setting, n (%)**					
	Single practitioner	72 (39.8)		1 (33)	
	Multiple dentists	109 (60.2)		2 (67)	
**Sex, n (%)**					
	Female	29 (16)		1 (33)	
	Male	72 (39.8)		1 (33)	
	Mixed	80 (44.2)		1 (33)	
**Dental society membership, n (%)**					
	No or unknown	69 (38.1)		1 (33)	
	Yes	112 (61.9)		2 (67)	
Dentists’ year of examination^a,b^, mean (SD)			1997.7 (9.9)		2003.0 (14.6)

^a^In the case of multiple dentists, the years of examination were averaged, if available.

^b^There were 9 missing values (ie, information regarding dentists’ years of examination was missing for all dentists of the respective private dental office).

### Websites

The median score regarding the technical and functional aspects (domain 1) was 64.3% (IQR 59.8%-69.0%; range 25.3%-82.1%). For this domain, the interrater reliability was 0.953, which indicates an excellent agreement [32]. Further details on the checklist’s items and results for each subdomain are shown in Multimedia Appendix 2. The median FRES was 40.0% (IQR 34.0%-49.0%; range 0%-64%). Therefore, the readability of most websites can be classified as “difficult” [38].

The median score regarding the comprehensiveness of information on dental erosion (domain 3) was 11.5% (IQR 3.9%-26.9%; range 0.0%-76.9%). For this domain, the interrater reliability was 0.922, which indicates an excellent agreement [44]. Further details regarding the checklist’s items and results for each subdomain are shown in Multimedia Appendix 3.

The median score regarding the generic quality (domain 4) was 16.7% (IQR 8.3%-23.3%; range 0.0%-53.3%). The interrater reliability of this domain was 0.923, which indicates an excellent agreement [44]. Further details regarding the checklist’s items and results for each subdomain are shown in Multimedia Appendix 4.

**Figure 2:**
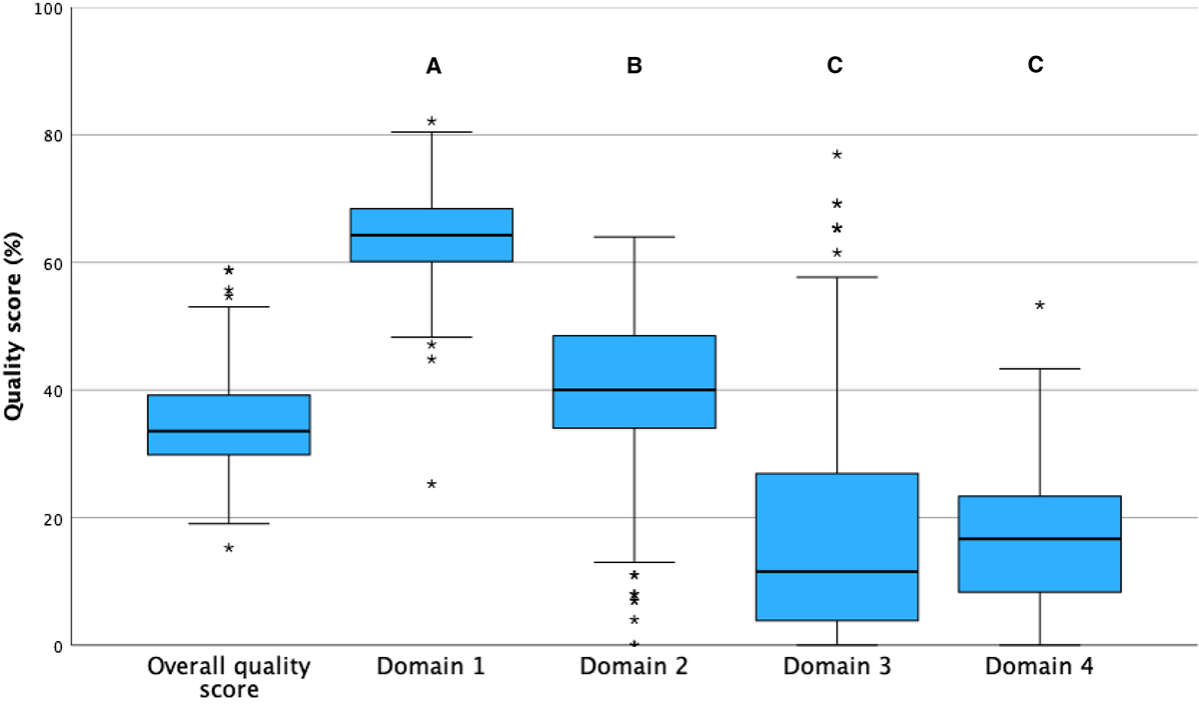
For all websites, the overall quality score (averaging the results from all 4 domains) and quality scores of each domain (relative percentage of maximum possible score sum) are shown. Domain 1: technical and functional aspects (LIDA instrument, version 1.2; Minervation); domain 2: readability (Flesch reading-ease score, adapted by Amstad for the German language); domain 3: comprehensiveness of information; domain 4: generic quality and risk of bias (DISCERN instrument). Significant differences between domains are marked by different letters (*P*<.001 for the Friedman test followed by Dunn-Bonferroni post hoc tests). Outliers are marked with an asterisk (*).

As a combination of all 4 domains, the overall quality score was 33.6% (IQR 29.8%-39.2%; range 15.3%-58.8%). Individual scores from different domains varied between domains (*χ*^2^_3_=517.7; *P*<.001 for the Friedman test) as shown in Figure 2. Domain 3 and domain 4 resulted in lower scores than domains 1 and 2 (both *P*_adjusted_<.001).

The overall quality score as well as individual scores from domain 1 and domain 3 varied between website providers (*P*≤.005 for Kruskal-Wallis rank-sum tests) as shown in Table 4. Websites from private dental offices resulted in lower overall quality scores, lower scores in domain 1, and lower scores in domain 3 than websites published by dental societies, dental regulatory bodies, public bodies, or insurance companies (*P*_adjusted_=.003; *P*_adjusted_<.001; *P*_adjusted_=.01, respectively). In addition, for domain 1, websites from information services resulted in lower scores than websites published by dental societies, dental regulatory bodies, public bodies, or insurance companies (*P*_adjusted_=.003).

**Table 4:** Quality scores of all domains and overall quality scores across different website providers.

Website provider	Overall quality score			Domain 1^a^			Domain 2^b^			Domain 3^c^			Domain 4^d^	
	Median (IQR)	Range	Median (IQR)		Range	Median (IQR)		Range	Median (IQR)		Range	Median (IQR)		Range
Private dental offices	33.3 (29.4-37.6)	15.3-58.7	63.2 (59.5-67.8)		25.3-80.5	40.0 (34.0-50.0)		0.0-64.0	7.7 (3.9-23.1)		0.0-69.2	16.1 (8.3-23.3)		0.0-53.3
Corporate dental offices or private hospital groups	33.4 (30.7-40.0)	19.6-48.1	66.1 (61.9-69.0)		57.5-73.6	40.0 (33.5-48.0)		0.0-53.0	13.5 (7.7-25.0)		3.9-53.9	12.1 (7.1-22.4)		5.0-41.1
Public dental clinics or dental schools	31.8 (26.0-40.2)	24.2-42.2	63.8 (60.9-69.0)		52.4-73.6	33.0 (24.0-43.0)		21.0-44.0	17.3 (3.9-34.6)		3.9-42.3	14.3 (7.1-21.7)		3.6-23.2
Dental societies, dental regulatory bodies, public bodies, or insurance companies	40.8 (34.2-50.4)	28.9-58.8	69.7 (67.2-75.3)		59.8-82.1	43.0 (40.3-50.5)		31.5-53.0	26.9 (13.5-51.9)		0.0-76.9	19.6 (10.3-36.2)		5.4-41.7
Information services	38.5 (34.3-40.0)	26.7-42.8	60.5 (57.9-64.2)		53.1-69.0	36.5 (31.0-38.0)		27.0-42.0	32.7 (17.3-40.4)		0.0-42.3	26.6 (19.6-30.4)		5.4-31.7
*P* value^e^	.005	—^f^	<.001		—	.09		—	.002		—	.11		—

^a^Technical and functional aspects (LIDA instrument, version 1.2; Minervation [37]).

^b^Readability (Flesch reading-ease score [38], adapted by Amstad [39] for the German language).

^c^Comprehensiveness of information.

^d^Generic quality and risk of bias (DISCERN instrument [40]).

^e^*P* values from Kruskal-Wallis rank-sum tests.

^f^Not applicable.

Regarding the relation between practice-specific variables of private dental offices and the overall quality score as well as individual scores from each domain, only domain 2 was associated with practice-specific variables as shown in Table 5. German-language websites from non-German private dental offices (*P*=.04 for GLM; B=–6.64, 95% CI –12.85 to –0.42) or from dentists who are a dental society member (*P*=.049 for GLM; B=–3.55, 95% CI –7.09 to –0.01) resulted in lower scores (ie, were more difficult to read), while a shorter time since examination resulted in higher scores (*P*=.01 for GLM; B=0.24 per year, 95% CI 0.05-0.43).

**Table 5:** Association between practice-specific variables and readability (Flesch reading-ease score) of websites provided by private dental offices. Model fit: χ^2^_8_=19.6; *P*=.01 (Likelihood ratio test).

Variable		Estimate B (95% CI)	*P* value
Foreign country (reference Germany)		–6.64 (–12.85 to –0.42)	.04
**Practice location**			
	Town (reference rural)	2.92 (–1.73 to 7.58)	.22
	City (reference rural)	2.18 (–2.39 to 6.74)	.35
Multiple dentists (reference single practitioner)		–0.67 (–5.81 to 4.47)	.80
**Sex**			
	Male only (reference female only)	–2.51 (–7.63 to 2.61)	.34
	Mixed (reference female only)	–1.14 (–7.29 to 5.00)	.72
Dental society membership (reference no or unknown)		–3.55 (–7.09 to –0.01)	.049
Dentists’ year of examination^a^		0.24 (0.05 to 0.43)	.01

^a^In the case of multiple dentists, the years of examination were averaged, if available.

### YouTube Videos

The median length of YouTube videos was 5.4 (IQR 4.0-7.1) minutes, and the median age was 2.8 (IQR 1.3-3.5) years. However, viewers’ interaction and viewing rates of YouTube videos were low. Further characteristics of included YouTube videos are shown in Table 6.

**Table 6:** Characteristics of included YouTube videos.

Variable	Value	
	Median (IQR)	Range
Age of video (years)	2.8 (1.3-3.5)	0.6-6.1
Duration (minutes)	5.4 (4.0-7.1)	0.5-10.8
Number of views	591 (523.5-1372.5)	105-2711
Number of likes	12 (6.5-18.5)	0-33
Number of dislikes	0 (0-0)	0-1
Number of comments	0 (0-1)	0-30
Comprehensiveness of information (%)	34.6 (13.5-38.5)	3.8-50.0
Viewers’ interaction (%)	1.71 (0.70-3.36)	0.00-4.04
Viewing rate (%)	101.0 (54.6-112.6)	23.5-525.0

Among YouTube videos, the median score regarding the comprehensiveness of information on dental erosion was 34.6% (IQR 13.5%-38.5%; range 3.8%-50.0%). Interrater reliability was 0.852, which indicates an excellent agreement [44]. However, the comprehensiveness of information did not vary between websites and YouTube videos (*P*=.09 for the Wilcoxon rank-sum test).

## Discussion

### Principal Findings

A total of 231 German-language websites and 7 YouTube videos containing patient-centered, web-based health information on erosive tooth wear were identified and included in the analysis. However, based on the total number of dentists in the DACH (Deutschland [Germany], Austria, and Confoederatio Helvetica [Switzerland]) countries, only a minority of dentists provide web-based health information on erosive tooth wear to (their) patients.

While most websites achieved sufficient scores regarding technical and functional aspects (median score 64.3%, IQR 59.8%-69.0%), the provided text presented a high reading difficulty (median score 40.0%, IQR 34.0%-49.0%). In addition, the comprehensiveness of information on dental erosion was low (median score 11.5%, IQR 3.9%-26.9%), and the generic quality was low resulting in a high risk of bias (median score 16.7%, IQR 8.3%-23.3%). For the calculation of an overall quality score (median score 33.6%, IQR 29.8%-39.2%), all 4 domains were assumed to be of equal importance, and individual domains’ quality scores were averaged. The overall quality score as well as individual scores from domain 1 and domain 3 varied between websites’ content providers. Thus, the first null hypothesis must be rejected.

Practice-specific variables of private dental offices (eg, years of examination and dental society membership) impacted information quality. As erosive tooth wear gained increased attention over the past decades, only dentists who graduated in recent years (ie, 10 to 15 years) can be expected to be educated about erosive tooth wear during their undergraduate dental curriculum. In addition, dentists who graduated recently are younger and possibly show a greater affinity for the web than older dentists. Dentists who are a dental society member can be expected to be more involved in continuing education and better informed about new developments. In addition, dental societies might spend more money on creating professional patient-centered information than individual dentists. The resulting information is likely of higher quality than self-made texts from individual dentists, and dental societies might provide the created information to their members to be shared with their patients (eg, on dental offices’ websites). As a result, dentists who graduated in recent years or dentists who are dental society members are more likely to provide information on erosive tooth wear to their patients and that information might be of higher quality. Regarding the effect of practice-specific variables of private dental offices on the overall quality score as well as individual scores, only scores from domain 2 were associated with practice-specific variables. Thus, the second null hypothesis must be rejected.

The number of available YouTube videos was limited. Among the assessed videos, comprehensiveness of information on dental erosion was also low (median score 34.6%; IQR 13.5%-38.5%). However, content providers of YouTube videos were limited to private dental offices, corporate dental offices, or private hospital groups. No YouTube videos published by public dental clinics, dental schools, dental societies, dental regulatory bodies, public bodies, or insurance companies were found.

### Comparison to Prior Work

Previous studies concerning the quality of web-based health information within the context of preventive and restorative dentistry either assessed websites [6,12,13,17,19-24] or YouTube videos [7-11,14-16,18] only. This study evaluated both formats of web-based health information on the same topic (ie, erosive tooth wear) and included a higher number of websites than the majority of the previously performed studies [6,12,13,17,19,20,22-24]. Furthermore, eligible sources were extended beyond websites published by dental offices. While patients in need of dental care are likely to primarily focus on dentists’ websites, patients might also find information on websites operated by public dental clinics or dental schools, dental societies, dental regulatory bodies, public bodies, insurance companies, or information services helpful to gain information and to participate in informed shared decision-making.

While some of the previous studies used different tools (ie, JAMA benchmarks, the presence of HONcode certification, and Global Quality Score), health information on endodontics, dental caries, restoration repair, and periodontitis resulted in overall LIDA scores between 55% and 72% [6,13,19,23], overall DISCERN scores between 4% and 61% [6,9-13,15,17,19,20,22,23], and overall FRES between 60% and 97% [12,13,17,20]. However, this study used the validated LIDA [37] and DISCERN [40] instruments as originally published instead of adjusting the assessment and scoring as it was done in previous studies within the context of preventive and restorative dentistry [9-11,15,19,23].

Some of the previous studies only assessed formal quality or readability [13,17,20-22]. In this study, the comprehensiveness of content-specific information regarding erosive tooth wear was also evaluated. Technical and functional aspects resulted in significantly higher scores than the comprehensiveness of information and generic quality. These findings are in line with previous studies, which also found lower scores regarding the comprehensiveness of the respective disease-specific information than scores regarding formal quality criteria [6,15,19,23]. However, a direct comparison between the scores on the comprehensiveness of information is not possible due to different checklists or qualitative analyses being applied in some studies.

### Practical Implications

Websites’ information quality on tooth wear was found to be limited. In addition, some information was scientifically inaccurate or misleading, and the available information was neither comprehensive nor trustworthy. As a result, dentists’ web-based information on erosive tooth wear requires improvement to inform patients comprehensively and reliably. Until then, patients should be aware of the limitations or gain information elsewhere.

This study found patient-centered, web-based health information on erosive tooth wear provided by dental societies, dental regulatory bodies, public bodies, or insurance companies as well as information services to be more comprehensive than information provided by dental offices. The quality of dentists’ web-based health information on erosive tooth wear might be increased by different interventions. First, higher information standards might be enforced by regulatory and legislative bodies. Second, dental societies, dental regulatory bodies, or public bodies might provide validated and high-quality content to dentists, which might be adopted by the individual dentists on their websites. Third, dentists might add links to external websites containing validated and high-quality content published elsewhere (eg, by dental societies, dental regulatory bodies, or public bodies).

### Strengths and Limitations

This study has several strengths increasing the overall reliability of our findings. First, websites were identified through a systematic search using 3 different consumer search engines. The selection of the used search engines (google.de, bing.de or yahoo.de, and duckduckgo.com) was based on the current usage data among German internet users at the time of this study [45]. Second, a high number of websites in total and websites operated by different service providers (eg, dental offices, dental societies, dental regulatory bodies, public bodies, insurance companies, and information services) were included. Third, 3 out of 4 domains were scored using established and validated tools [37,38,40]. Fourth, all domains that required manual scoring were independently assessed by 2 different raters. Interrater reliability was found to be excellent (≥0.852) throughout all domains [44]. Fifth, technical and functional aspects were tested using a variety of browsers (Google Chrome, Firefox, Microsoft Edge, and Apple Safari). Again, the selection of the browsers was based on their current usage frequency among German internet users at the time of this study [46].

However, several limitations are present. First, only websites and YouTube videos published in German were assessed. Websites in other languages might show a higher quality or divergent results. However, previous studies regarding the quality of websites within the context of periodontitis found a low quality of information for both German- and English-language websites [21,23]. Second, most websites were published by service providers located in the DACH countries. Therefore, the transferability and generalizability of the findings to other countries or health care systems are unknown. Third, the comprehensiveness of information on dental erosion was assessed using a checklist developed by the authors. As this checklist was not formally tested, a different checklist might have yielded different results. Fourth, only the comprehensiveness of information regarding dental erosion but not its scientific accuracy was evaluated. For example, numerous websites recommended a waiting time between the consumption of erosive food or drinks and toothbrushing, but current evidence suggests that delayed toothbrushing is not capable of preventing erosive tooth wear [47]. Fifth, while the accessibility and usability of websites were assessed using the LIDA instrument, no dedicated tools focusing on accessibility to people with disabilities (eg, Web Content Accessibility Guidelines 2.0) were used.

### Future Directions

More dentists should offer web-based information on erosive tooth wear to (their) patients. In addition, the content should be more comprehensive, trustworthy, and of higher quality than the currently available web-based health information on erosive tooth wear. To increase the number of reliable websites, dental societies, dental regulatory bodies, or public bodies might assist dentists by providing high-quality content for further adaptation by the individual dentist.

### Conclusions

The quality of German-language, patient-centered, web-based information on erosive tooth wear was found to be limited. The available information was neither comprehensive nor trustworthy. However, quality differed between content providers, and practice-specific variables impacted the readability of websites from private dental offices. Web-based information on erosive tooth wear requires improvement to inform patients comprehensively and reliably and to allow for patients’ participation in informed shared decision-making.

## Appendix

Multimedia Appendix 1 List of included websites and YouTube videos with information on erosive tooth wear and dental erosion.

Multimedia Appendix 2 Results for each subdomain regarding technical and functional aspects (domain 1: LIDA instrument, version 1.2; Minervation).

Multimedia Appendix 3 Results for each subdomain regarding the comprehensiveness of information on dental erosion (domain 3).

Multimedia Appendix 4 Results for each subdomain regarding generic quality and risk of bias (domain 4: DISCERN instrument).

## Abbreviations

DACH: Deutschland (Germany), Austria, and Confoederatio Helvetica (Switzerland)
DGZMK: Deutsche Gesellschaft für Zahn-, Mund- und Kieferheilkunde (German Society of Dentistry and Oral Medicine)
ENTREQ: Enhancing Transparency in Reporting the Synthesis of Qualitative Research
FRES: Flesch reading-ease score
GLM: generalized linear modeling
PRISMA: Preferred Reporting Items for Systematic Reviews and Meta-Analyses
SSO: Swiss Dental Association

## Data Availability

All data generated during or analyzed during this study are included in this published article and its supplementary information files.
