# Supplementary material for: Quality of Patient-Centered eHealth Information on Erosive Tooth Wear: Systematic Search and Evaluation of Websites and YouTube Videos
Source: J Med Internet Res. 2024 Jan 31;26:e49514. doi: 10.2196/49514 (PMC10867746; doi:10.2196/49514)
Supplement: Multimedia Appendix 1 [file jmir_v26i1e49514_app1.doc]

**Multimedia Appendix 1: List of included websites and YouTube videos with information on erosive tooth wear and dental erosion.**

| **Content provider’s name** | **URL** | **Country** | **Location^a^** | **Content provider^b^** | **Dental society membership** | **Year of examination** |
| --- | --- | --- | --- | --- | --- | --- |
| Zahnarzt Gelsenkirchen, Dr. Michael Crass | <https://www.zahnarztpraxis-crass.de/media/shop/layout/home/downloads-TippszurzahngesundenErnaehrung.pdf> | Germany | Gelsenkirchen (city) | Private dental office, single practitioner (male) | DGZMK, DGI, DGParo, FVDZ | 1986 [1]^c^ |
| Zahnarztpraxis am Landratsplatz; Dr. Jens Nolte | <https://www.drnolte.de/aktuelles-1> | Germany | Bad Segeberg (town) | Private dental office, single practitioner (male) | DGZMK, DGParo, DGÄZ | 1992 |
| Zahnarztpraxis Dr. Plehwe | [https://www.zahnarzt-plehwe.de/information/karies-ern%C3%A4hrung/](https://www.zahnarzt-plehwe.de/information/karies-ernährung/) | Germany | Saarbrücken (city) | Private dental office, single practitioner (male) | DGZMK, BDIZ, DGZC | 1987 [2]^c^ |
| Zahnarztpraxis Neu Fahrland; Regine Boettcher | <https://www.potsdam-zahnarzt.com/aktuelles/gesundheit-medizin/artikel/14899.pdf> | Germany | Potsdam (city) | Private dental office, single practitioner (female) | – | 1970 |
| Zahnarztpraxis Ernst-Walter Vollmer | [https://www.zahnmedizin-prophylaxe-rethem.de/1-212-516-Zahnm%C3%A4nnchen-zur-Kariespr%C3%A4vention-ist-Siegel-der-Aktion-zahnfreundlich.html](https://www.zahnmedizin-prophylaxe-rethem.de/1-212-516-Zahnmännchen-zur-Kariesprävention-ist-Siegel-der-Aktion-zahnfreundlich.html) | Germany | Rethem (town) | Private dental office, single practitioner (male) | – | 2001 |
| Praxis für Zahngesundheit; Dr. Uhlig | [https://www.zahngesundheit-hamburg.de/1-212-516-Zahnm%C3%A4nnchen-zur-Kariespr%C3%A4vention-ist-Siegel-der-Aktion-zahnfreundlich.html](https://www.zahngesundheit-hamburg.de/1-212-516-Zahnmännchen-zur-Kariesprävention-ist-Siegel-der-Aktion-zahnfreundlich.html) | Germany | Hamburg (city) | Private dental office, single practitioner (male) | BDIZ | 1973 [3]^c^ |
| Zahnarztpraxis Dr. Thiem-Müller | <https://www.zahnarzt-thiem-mueller-witten.de/praxis/aktuelles.html> | Germany | Witten (town) | Private dental office, single practitioner (female) | FVDZ | 1991 [4]^c^ |
| Doc Grosch; Dr. Grosch | [https://www.doc-grosch.de/1-212-516-Zahnm%C3%A4nnchen-zur-Kariespr%C3%A4vention-ist-Siegel-der-Aktion-zahnfreundlich.html](https://www.doc-grosch.de/1-212-516-Zahnmännchen-zur-Kariesprävention-ist-Siegel-der-Aktion-zahnfreundlich.html) | Germany | Coburg (town) | Private dental office, single practitioner (male) | DGZMK, DGK, DGParo | 1985 [5]^c^ |
| Zahnarztpraxis MUNDwerk-Wadersloh; Dr. Steinhoff | <https://www.mundwerk-wadersloh.de/aktuelles/zahnschaeden-so-aetzend-sind-energydrinks/index.html> | Germany | Wadersloh (rural) | Private dental office, single practitioner (male) | DGZI, ICOI | 1995 [6]^c^ |
| Rosenpraxis; Dr. Sven Hotz | <https://www.rosenpraxis.de/faqs/> | Germany | Rosenfeld (town) | Private dental office, single practitioner (male) | DZOI, BDIZ | 1993 |
| Zahnarztpraxis Dr. Malert | <https://www.zahn-arzt-oberhausen.de/glossar/versiegelung/> | Germany | Oberhausen (city) | Private dental office, single practitioner (male) | DGParo, DGET, BDIZ, DGZI | 1981 [7]^c^ |
| Zahnarztpraxis Klabunde | <https://www.praxis-klabunde.de/index.php/blog/item/12-bulimie> | Germany | Osterholz-Scharmbeck (town) | Private dental office, single practitioner (male) | – | 1995 [8]^c^ |
| Zahnarztpraxis Jensen | [https://www.ihr-zahnarzt-jensen.de/1-212-516-Zahnm%C3%A4nnchen-zur-Kariespr%C3%A4vention-ist-Siegel-der-Aktion-zahnfreundlich.html](https://www.ihr-zahnarzt-jensen.de/1-212-516-Zahnmännchen-zur-Kariesprävention-ist-Siegel-der-Aktion-zahnfreundlich.html) | Germany | Risum-Lindholm (rural) | Private dental office, single practitioner (male) | DGZMK, DGCZ | 1996 |
| Zahnarztpraxis Dahlke-Kragelund | [https://www.zahnarztpraxis-grossenwiehe.de/1-212-516-Zahnm%C3%A4nnchen-zur-Kariespr%C3%A4vention-ist-Siegel-der-Aktion-zahnfreundlich.html](https://www.zahnarztpraxis-grossenwiehe.de/1-212-516-Zahnmännchen-zur-Kariesprävention-ist-Siegel-der-Aktion-zahnfreundlich.html) | Germany | Großenwiehe (rural) | Private dental office, single practitioner (female) | – | 1990 |
| Zahnarztpraxis Rosenwald | [https://www.zahnarztpraxis-rosenwald.de/1-212-516-Zahnm%C3%A4nnchen-zur-Kariespr%C3%A4vention-ist-Siegel-der-Aktion-zahnfreundlich.html](https://www.zahnarztpraxis-rosenwald.de/1-212-516-Zahnmännchen-zur-Kariesprävention-ist-Siegel-der-Aktion-zahnfreundlich.html) | Germany | Husum (town) | Private dental office, single practitioner (female) | – | N/A |
| AAA Ästhetische Zahnmedizin; Dr. Gaßmann | <https://www.drgassmann.de/blog/select_category/5.html>  <https://www.drgassmann.de/blog/post/article/zahnschaeden-durch-ess-stoerungen.html> | Germany | Hamburg (city) | Private dental office, single practitioner (male) | DGZMK, DGI, DZOI, BDIZ, DGCZ, DGKFO | 1988 |
| Zahnarzt Dr. Lessing | [http://www.dr-lessing.de/zahnvorsorge/#](http://www.dr-lessing.de/zahnvorsorge/)  <http://www.dr-lessing.de/zahnvorsorge/> | Germany | Friburgh (city) | Private dental office, single practitioner (male) | DGParo, DGZI | 1989 [9]^c^ |
| Praxis für Zahngesundheit; Dr. Kettler-Nölke | [https://www.zahnarztpraxis-hamburg-niendorf.de/1-212-516-Zahnm%C3%A4nnchen-zur-Kariespr%C3%A4vention-ist-Siegel-der-Aktion-zahnfreundlich.html](https://www.zahnarztpraxis-hamburg-niendorf.de/1-212-516-Zahnmännchen-zur-Kariesprävention-ist-Siegel-der-Aktion-zahnfreundlich.html) | Germany | Hamburg (city) | Private dental office, single practitioner (female) | DGZMK, DGI, DGCZ, AG Keramik, FVDZ | 1973 [10]^c^ |
| Zahnarztpraxis Dr. Streletz | <https://www.dr-streletz.de/praxis/prophylaxe/> | Germany | Heusenstamm (town) | Private dental office, single practitioner (female) | DGParo, NAgP | 1985 [11]^c^ |
| Zahnarzt Dr. Seidel | <https://www.zahnarzt-drseidel.de/essstoerung-bulimie.html>  <https://www.zahnarzt-drseidel.de/news/zahnerosion.html>  <https://www.zahnarzt-drseidel.de/abrasion.html> | Germany | Kleinmachnow (rural) | Private dental office, single practitioner (male) | BDIZ, DGI, DGZI, ICOI, BDO, FVDZ | 1992 [12]^c^ |
| Zahnarztpraxis Dr. Deisenhofer | <https://www.zahnarztpraxis-deisenhofer.de/zahnschaeden/> | Germany | Adelsried (rural) | Private dental office, single practitioner (male) | – | 1992 [13]^c^ |
| Zahnarztpraxis Bosch und Partner | [https://www.konstanz-zahnarzt.de/gesundheit/saeureschaeden-zahnschmelz/#](https://www.konstanz-zahnarzt.de/gesundheit/saeureschaeden-zahnschmelz/)  <https://www.konstanz-zahnarzt.de/gesundheit/saeureschaeden-zahnschmelz/> | Germany | Constance (town) | Private dental office, single practitioner (male) | – | N/A^d^ |
| Zahnarztpraxis Dr. Weyland | <https://zahnarzt-weyland.de/wp-content/uploads/2015/07/Weyland_Zahnersatz.pdf> | Germany | Riegelsberg (rural) | Private dental office, single practitioner (male) | DGZMK | 2001 [14]^c^ |
| Praxis Dr. Graeber | <https://drgraeber.de/aktuelles/12-warum-energy-drinks-unsere-zaehne-kaputt-machen> | Germany | Brunswick (city) | Private dental office, single practitioner (male) | – | 1990 |
| Nordseepraxis; ZÄ Tönissen | <https://www.nordseepraxis.de/GrosseKinder.pdf> | Germany | Buchholz Nordheide (town) | Private dental office, single practitioner (female) | DGZMK, DGCZ^e^ | 1998 [15]^f^ |
| Zahnarztpraxis Weller | <https://zahnarzt-weller.de/zahnerhaltung.html> | Germany | Beckingen-Düppenweiler (rural) | Private dental office, single practitioner (male) | – | 1997 |
| Zahnarztpraxis Dr. Spiekermann | <https://spiekermann-zahnarztpraxis.de/blog/>  (<https://spiekermann-zahnarztpraxis.de/blog/mundgesundheit-und-schwangerschaft/>) | Germany | Stralsund (town) | Private dental office, single practitioner (male) | – | 2015 [16]^g^ |
| Zahnarztpraxis Schulz | <https://www.zahnarzt-schulz-heidelberg.de/gesundheitsnews/> | Germany | Heidelberg (city) | Private dental office, single practitioner (male) | – | N/A |
| Zahnarztpraxis am Löhrs Carré; Dr. Tegeler | <https://www.zahnarztpraxis-leipzig-zentrum.de/rund-um-ihre-zaehne/gesundheitsnews/> | Germany | Leipzig (city) | Private dental office, single practitioner (female) | – | 2005 |
| Zahnarztpraxis Radwan | <https://www.zahnarztpraxis-radwan.de/gesundheitsnews/> | Germany | Windhagen (rural) | Private dental office, single practitioner (male) | – | N/A |
| Zahnarztpraxis Blumentrath | <https://www.mein-zahnarzt-ellwangen.de/news/> | Germany | Ellwangen/Jagst (town) | Private dental office, single practitioner (male) | – | 1998 |
| Zahnarztpraxis Dr. Diekmeier | <https://www.zahnarzt-diekmeier.de/gesundheitsnews/> | Germany | Hörstel-Riesenbeck (town) | Private dental office, single practitioner (male) | – | 1993 [17]^c^ |
| Zahnarztpraxis Stoltenow | <https://www.zahnarzt-stoltenow-beelitz.de/gesundheitsnews> | Germany | Beelitz (town) | Private dental office, single practitioner (male) | – | 1984 [18]^g^ |
| Zahnarztpraxis Wolski | <https://zahnarzt-wolski.de/neuigkeiten-in-der-uebersicht/94-gefaehrlicher-abrieb-der-zaehne> | Germany | Munich (city) | Private dental office, single practitioner (female) | DGZMK, DGZ, DGL, FVDZ | N/A |
| Implanteer®; Dr. Hinze und Kollegen | <https://www.implanteer.de/blog/zahnerosion-zahnsubstanz-geht-durch-saeureattacken-verloren-wirkung-von-getraenken-nahrungsmitteln-und-magensaeure/>  <https://www.implanteer.de/blog/tag/zahnerosion/> | Germany | Gräfelfing (town) | Private dental office, single practitioner (male) | DGZMK, DGI, DGParo^e^ | 2005 |
| Zahnarztpraxis Mirzakhanian | <https://www.zahnarzt-oststeinbek-bergedorf.de/2017/07/21/sensible-zaehne-brauchen-pflege-und-effektiven-schutz/> | Germany | Oststeinbeck (rural) | Private dental office, single practitioner (male) | DGZMK, DGCZ | 2004 |
| Zahnarztpraxis Dr. Steinigen | <https://za-steinigen.de/neuigkeiten-wissenswertes/> | Germany | Pirna (town) | Private dental office, single practitioner (female) | – | N/A |
| Zahnarztpraxis Otto | <https://www.zahnarzt-suderburg.de/> | Germany | Suderburg (rural) | Private dental office, single practitioner (male) | – | 1981 |
| Praxis für Zahnheilkunde; Dr. Hauptmann | <https://www.zahnarzt-hauptmann.de/gesundheitsnews/> | Germany | Neustadt (town) | Private dental office, single practitioner (male) | DGZMK, DGI, DGÄZ | 1998 |
| Zahnarztpraxis Dr. Mönch | <https://zahnarzt-moench.de/o-saft-schadet-zaehnen.html> | Germany | Berlin (city) | Private dental office, single practitioner (female) | – | 1987 [19]^c^ |
| Zahnarztpraxis Dr. Königer | https://www.zahnarzt-wassertrüdingen.de/gesundheitsnews/ | Germany | Wassertrüdingen (town) | Private dental office, single practitioner (male) | – | 2005 [20]^c^ |
| Zahnarztpraxis am Schloss; Dr. Rodiger | <https://www.zahnarztpraxis-rodiger.de/prophylaxe/> | Germany | Rastatt (town) | Private dental office, single practitioner (male) | DGZMK, DGParo | 1997 |
| Team für Zahnmedizin; Dr. Dummler | <https://www.team-fuer-zahnmedizin.de/>  <https://www.team-fuer-zahnmedizin.de/leistungsspektrum/prophylaxe/erosionen/> | Germany | Hamburg (city) | Private dental office, single practitioner (female) | – | 1995 [21]^c^ |
| Praxis für Zahnheilkunde; Dr. Kappl | <https://www.zahnarzt-dr-kappl.de/erosionen.html>  <https://www.zahnarzt-dr-kappl.de/behandlungsspektrum.html> | Germany | Filderstadt (town) | Private dental office, single practitioner (male) | DGZMK, DGParo, APW^e^ | 1981 |
| Zahnarztpraxis Gebhardt | <https://www.zahnarztpraxis-gebhardt.net/zahnmed-leistungen/> | Germany | Berlin (city) | Private dental office, single practitioner (male) | DGZMK, DGI | 2005 |
| Zahnarztpraxis Dr. Borchard | <http://www.entspannt-beim-zahnarzt.de/fallbeispiele/veneers/index.php> | Germany | Münster (city) | Private dental office, single practitioner (male) | – | 1986 [22]^c^ |
| Zahnarztpraxis Willus | <https://www.zahnarzt-willus.de/prophylaxe_pzr.php> | Germany | Munich (city) | Private dental office, single practitioner (male) | DGKZ, ESCD | 1976 |
| Praxis für Zahnerhaltung; Dr. Brandt | <https://www.zahnarzt-kelkheim.dentist/de/zahnarzt-kelkheim-informatives/> | Germany | Kelkheim (town) | Private dental office, single practitioner (male) | – | 1996 [23]^c^ |
| Zahnarztpraxis Dr. Krettek | <https://www.prophylaxe-in-lehrte.de/gesundheitsnews/> | Germany | Lehrte (town) | Private dental office, single practitioner (female) | DGZMK^e^ | 1990 [24]^c^ |
| Praxis für dentale Ästhetik; Dr. Hayim | <https://www.cosmetic-dental.de/zahnerosion-behandlung/> | Germany | Essen-Rüttenscheid (town) | Private dental office, single practitioner (male) | DGZMK, DGI, APW | 1990 |
| Zahnarztpraxis Padilla | <https://www.ap-zahnarzt-frankfurt.de/info/dentale-erosionen/> | Germany | Frankfort-on-the-Main (city) | Private dental office, single practitioner (male) | DGZMK, DGI, DGParo, DGET, DGÄZ | 2001 |
| Gesundes Lächeln; Dr. Fremgen | <https://www.gesundes-laecheln.de/zahnschmelzerosion-durch-gesunde-ernaehrung/>  <https://www.gesundes-laecheln.de/zahnerosion-eine-herausforderung-fur-die-zahngesundheit/> | Germany | Glan-Münchweiler (rural) | Private dental office, single practitioner (female) | – | 1984 [25]^c^ |
| Zahnarztpraxis Dr. Kendzia | <https://www.dr-kendzia.de/glossar-dr-kendzia/211-erosion-des-schmelzes.html>  <https://www.dr-kendzia.de/images/stories/infobroschueren/p-z/Zahn-Erosion_2012.pdf> | Germany | Fußgönnheim (rural) | Private dental office, single practitioner (male) | DGZMK, DGAZ, DGCZ^e^ | 1985 |
| Zahnarzt Dr. Ullrich | <https://www.dr-stefan-ullrich.de/funktionelle-stoerungen/abrasionserosionen> | Germany | Weiden (town) | Private dental office, single practitioner (male) | DGZMK, DGI, DGParo, DGÄZ, DGET, BdiZ, EAO, EDA, APW, DGZS, DGFDT^e^ | 1993 |
| Zahnarzt Dr. Schleenbecker | <https://www.blog.dr-schleenbecker.de/erosionen/> | Germany | Berg (rural) | Private dental office, single practitioner (male) | DGZMK, DGFDT, DGOI, DGZH, EDA, DGZSM | 1997 |
| Zahnarztpraxis Dr. Riegger | <http://www.zahnarzt-riegger.de/wissenswertes_erosion.html> | Germany | Albstadt (town) | Private dental office, single practitioner (male) | DGZMK, APW^e^ | 1983 [26]^c^ |
| Aesthetic smile; Sabine Bormann | <https://www.aesthetic-smile-husum.de/leistungsspektrum/zahnerhaltung/erosionen/> | Germany | Husum (town) | Private dental office, single practitioner (female) | DGZMK, DGParo, FVDZ | 2008 |
| Zahnarzt Keiss Woldegk; Dr. Keiss | <https://www.zahnarzt-keiss-woldegk.de/> | Germany | Woldegk (rural) | Private dental office, single practitioner (female) | – | 2004 [27]^c^ |
| Zahnarztpraxis Dr. Tauber | <https://www.dr-tauber.com/gesundheitsnews/> | Germany | Munich (city) | Private dental office, single practitioner (male) | DGI | 1996 |
| Zahnarzt im Theresientor; ZA Binner | <https://www.zahnarzt-binner-straubing.de/leistungen/prophylaxe/> | Germany | Straubing (town) | Private dental office, single practitioner (male) | LAGZ | 1986 |
| Zahnarztpraxis Dr. Dobl | <http://www.zahnarztpraxis-dr-dobl.de/lexikon.php?id=192> | Germany | Oelsnitz (town) | Private dental office, single practitioner (female) | – | 1986 |
| Zahnarztpraxis am Rathaus, ZÄ Schramm | <https://zahnarztpraxis-am-rathaus.de/behandlung/> | Germany | Kirchentellinsfurt (rural) | Private dental office, single practitioner (female) | – | 1997 |
| FitDentist; Dr. Rahimian | <https://www.fitdentist.de/leistungen/sportzahnmedizin> | Germany | Hamburg (city) | Private dental office, single practitioner (female) | DGZMK | N/A |
| Zahnarztpraxis Dr. Seitz-Klucenty | https://www.zahnärztin-dr-seitz.de/erosionen | Germany | Stuttgart (city) | Private dental office, single practitioner (female) | DGÄZ, DGET, FVDZ | 1985 |
| Zahnarztpraxis Dr. Speer | <https://www.dr-speer.de/fuellungstherapie/> | Germany | Bielefeld (city) | Private dental office, single practitioner (male) | – | 1993 |
| Zahnarztpraxis Hofmann | <https://zahnarzthofmann.de/sind-kaugummis-gesund/> | Germany | Königsbrück (town) | Private dental office, single practitioner (male) | – | 2006 |
| Zahnarztpraxis Kohl | <http://zahnarzt-oliver-kohl.de/zahnerhaltung/prophylaxe.html> | Germany | Dinslaken (town) | Private dental office, single practitioner (male) | – | N/A |
| Zahnarztpraxis Aeschbacher | <https://www.zahnarztpraxis-aeschbacher.ch/angebot> | Foreign (Switzerland) | Herzogenbuchsee (rural) | Private dental office, single practitioner (male) | SSO | 1982 [28]^c^ |
| Zahnarztpraxis Dr. Meyer | <https://www.zahnarzt-teresameyer.ch/erosionen-ein-zunehmendes-zahnproblem/> | Foreign (Switzerland) | Zurich (city) | Private dental office, single practitioner (female) | SSO | 2005 [29]^c^ |
| Zahnarztpraxis Dr. Faissler | <http://www.ihr-zahni.ch/index.php/aktuell>  (<http://www.ihr-zahni.ch/index.php/aktuell/37-merkblatt>) | Foreign (Switzerland) | Adliswil (town) | Private dental office, single practitioner (male) | SSO, SGI-SSIO | 1988 [30]^g^ |
| Zahnarztpraxis Dr. Bonatesta | <https://www.bonatesta.ch/> | Foreign (Switzerland) | Zurich (city) | Private dental office, single practitioner (male) | SSO | 1994 [31]^c^ |
| Zahnarzt Dr. Schmielau | <http://www.zahnarzt-innsbruck.at/body-food.html> | Foreign (Austria) | Innsbruck (city) | Private dental office, single practitioner (male) | ÖGZMK | 2005 |
| Gemeinschaftspraxis Dr. Pink, Dr. Wolferstätter und Kollegen | <https://www.max34.de/frauen-schlechtere-zaehne-als-maenner-2/> | Germany | Munich (city) | Private dental office, multiple dentists (two male dentists) | DGZMK, DGI, DGParo, DGZ, DGET, IGÄM, DGÄZ | 2001, 2011 |
| QUARREE DENTAL, Gemeinschaftspraxis für Zahnheilkunde | <https://quarree-dental.de/zahnprophylaxe-hamburg-wandsbek/> | Germany | Hamburg (city) | Private dental office, multiple dentists (four male, one female dentist) | DGZMK, DGI, DGParo, DGET, DGÄZ, DEGUZC, DGzPRSport, DGCZ | 1982, 2007, 2008, 2014, 1991 [32]^f^ |
| Zahnärzte am Marienplatz; Dres. Hoischen und Kelch | <https://www.zahnaerzte-marienplatz.de/funktionsschiene-bei-cranio-mandibulaerer-dysfunktion-cmd/> | Germany | Munich (city) | Private dental office, multiple dentists (two male dentists) | BDIZ, DGZS | 2005 [33]^c^, 2014 |
| Zahnarztpraxis Dres. Thomsen und Kollegen | <https://dr-thomsen.com/abrasionsgebiss> | Germany | Hamburg (city) | Private dental office, multiple dentists (three male, one female dentist) | DGZMK, DGI, DGParo, DGCZ, DGZS, DGÄZ, APW, AGOKI^a^ | 1974 [34]^c^ , 2009 [35]^c^, 1996, N/A |
| Zahnärzte am Lutherplatz; Dr. Greßbach und Kollegen | <https://kinderzahnarzt-lutherplatz.de/vorsorge/> | Germany | Langen (town) | Private dental office, multiple dentists (three female, one male dentist) | DGZMK, DGI, DGParo, BDIZ, BDO, DGZI, DGMKG, DGET, DGKiz | 2012 [36]^c^, 2009, 2017 [37]^f^, N/A |
| Zahnarztpraxis Dr. Seibold | <https://www.zahnarztpraxis-seibold.de/news/news-2020/april-2020/>  <https://www.zahnarztpraxis-seibold.de/news/news-2019/juni-2019/> | Germany | Stuttgart (city) | Private dental office, multiple dentists (two female dentists) | – | 1981 [38]^c^, N/A |
| Zahnarztpraxis Westend | <https://www.zahnarztpraxis-westend.com/media/shop/layout/home/080909_westend_downloads_zahngesundeErnaehrung.pdf> | Germany | Berlin (city) | Private dental office, multiple dentists (four male, three female dentists) | DGZMK, DGI, DGOI, BGP, BDI, DGFDT, FVDZ, APW^e^ | 1987, 1996, 2013, 2015, 1987, 2014, N/A |
| Zahnarztpraxis Dres. Evers-Lang und Lang | [https://www.apfelzahn.de/1-212-516-Zahnm%C3%A4nnchen-zur-Kariespr%C3%A4vention-ist-Siegel-der-Aktion-zahnfreundlich.html](https://www.apfelzahn.de/1-212-516-Zahnmännchen-zur-Kariesprävention-ist-Siegel-der-Aktion-zahnfreundlich.html) | Germany | Karlstein und Großkrotzenburg (rural) | Private dental office, multiple dentists (two female, one male dentist) | – | 1996 [39]^c^, 1996, 2015 |
| Praxis für Zahnheilkunde; Dr. Baitz und Kollegen | [https://www.dr-baitz.de/1-212-516-Zahnm%C3%A4nnchen-zur-Kariespr%C3%A4vention-ist-Siegel-der-Aktion-zahnfreundlich.html](https://www.dr-baitz.de/1-212-516-Zahnmännchen-zur-Kariesprävention-ist-Siegel-der-Aktion-zahnfreundlich.html) | Germany | Eckernförde (town) | Private dental office, multiple dentists (two female dentists) | – | 2004, 2017 |
| Zahnärzte an der Kleinmachnower Schleuse; Prof. Naumann, Dr. Kiessling und Kollegen | <https://www.naumann-kiessling.de/prophylaxe/> | Germany | Stahnsdorf (rural) | Private dental office, multiple dentists (two female, one male dentist) | DGZMK, DGI, DGParo, DGPro, DGFDT, APW, ITI, IADR, AAP, DGÄZ | 1998 [40]^c^, 1998 [41]^c^, 2012 [42]^c^ |
| Zahnarztpraxis im Fürsthof; Dr. Hinsche | <https://www.zahnarzt-hinsche.de/infos/zahnarzt-deutsch/> | Germany | Neumünster (town) | Private dental office, multiple dentists (two female dentists) | DGZMK, DGI, DGÄZ, DGQZ | 1999, N/A |
| Dr. Basset | <https://zahnmedizin-zentrum-radolfzell.de/youth-club/> | Germany | Radolfzell (town) | Private dental office, multiple dentists (two male, one female dentist) | DGZMK, DGI, DGÄZ, APW^e^ | 1991 [43]^c^, 2015 [44]^c^, 2016 |
| Zahnarztpraxis Dr. Lempa | <https://www.zahnarzt-wolfenbuettel.de/bisshebungen/> | Germany | Wolfenbüttel (town) | Private dental office, multiple dentists (two female, one male dentist) | DGOI, ICOI | 1999 [45]^c^, 2000, 1998 |
| Zahnarzt Pook | [https://www.zahnarzt-holm.de/1-212-516-Zahnm%C3%A4nnchen-zur-Kariespr%C3%A4vention-ist-Siegel-der-Aktion-zahnfreundlich.html](https://www.zahnarzt-holm.de/1-212-516-Zahnmännchen-zur-Kariesprävention-ist-Siegel-der-Aktion-zahnfreundlich.html) | Germany | Holm (rural) | Private dental office, multiple dentists (one female, one male dentist) | DGZMK | 1994, 2014 |
| Zahnärzte im Kronprinzkarree; Dres. Bethig, Heldt und van der Laden | <https://www.zahnarzt-bethig.de/leistungen/aesthetik/bisshebungen.html> | Germany | Berlin (city) | Private dental office, multiple dentists (two male, one female dentist) | – | 2016 [46]^c^, 2014 [47]^c^, N/A |
| Praxen für Zahngesundheit; Dr. Krauß, Dr. Hoffmann und Heldmann | [https://zahnarzt-prophylaxe-kiel.de/patienteninformation/1-212-516-Zahnm%C3%A4nnchen-zur-Kariespr%C3%A4vention-ist-Siegel-der-Aktion-zahnfreundlich.html/](https://zahnarzt-prophylaxe-kiel.de/patienteninformation/1-212-516-Zahnmännchen-zur-Kariesprävention-ist-Siegel-der-Aktion-zahnfreundlich.html/) | Germany | Kiel (city) | Private dental office, multiple dentists (five male, one female dentist) | – | 1994 [48]^c^, 1994, 2003 [49]^c^, 2011, 2017, 2019 |
| Praxis für ganzheitliche Zahnheilunde; Dr. Schumann und Kollegen | <http://www.ammersee-zahnaerzte.de/wissen> | Germany | Utting (rural) | Private dental office, multiple dentists (one female, one male dentist) | DGZMK, DGZ, FVDZ | 1990, 2019 |
| Zahnarztpraxis Dr. Imberg und Kollegen | [https://www.zahnarzt-imberg.de/1-212-516-Zahnm%C3%A4nnchen-zur-Kariespr%C3%A4vention-ist-Siegel-der-Aktion-zahnfreundlich.html](https://www.zahnarzt-imberg.de/1-212-516-Zahnmännchen-zur-Kariesprävention-ist-Siegel-der-Aktion-zahnfreundlich.html) | Germany | Gifhorn (town) | Private dental office, multiple dentists (one female, one male dentist) | DGZI, DGZS | 2003 [50]^c^, N/A |
| Zahnarztpraxis Dr. Hinrichsen | [https://www.zahnarzt-kiel-mitte.de/56-212-516-Zahnm%C3%A4nnchen-zur-Kariespr%C3%A4vention-ist-Siegel-der-Aktion-zahnfreundlich.html](https://www.zahnarzt-kiel-mitte.de/56-212-516-Zahnmännchen-zur-Kariesprävention-ist-Siegel-der-Aktion-zahnfreundlich.html) | Germany | Kiel (city) | Private dental office, multiple dentists (two male dentists) | DGI | 1994, 2015 |
| Zahnarzt Leverkusen; Dr. Kappek | <https://www.zahnarzt-in-leverkusen.de/media/shop/layout/home/dr-berthold-kappek-praxis-ernaehrung.pdf> | Germany | Leverkusen (city) | Private dental office, multiple dentists (two male, one female dentist) | – | 2002, 2013, 2002 |
| Zahnärztezentrum Münser; Dr. Wietzorke und Kollegen | <https://www.dr-wietzorke.de/blog/artikel/gesunde-ernaehrung-gesunde-zaehne> | Germany | Münster (city) | Private dental office, multiple dentists (three male, one female dentist) | – | 1985, 2013, 2021, 2019 |
| Zahnarztpraxis Gehring | [https://www.zahnarzt-oyten.de/1-212-516-Zahnm%C3%A4nnchen-zur-Kariespr%C3%A4vention-ist-Siegel-der-Aktion-zahnfreundlich.html](https://www.zahnarzt-oyten.de/1-212-516-Zahnmännchen-zur-Kariesprävention-ist-Siegel-der-Aktion-zahnfreundlich.html) | Germany | Oyten (town) | Private dental office, multiple dentists (one female, one male dentist) | DGZMK, DGI, AGOKI^e^ | 1997, 1999 |
| Praxis für Zahngesundheit und Prophylaxe; Dres. Lau und Priepke | [https://www.zahnarztpraxis-berlin-treptow.de/1-212-516-Zahnm%C3%A4nnchen-zur-Kariespr%C3%A4vention-ist-Siegel-der-Aktion-zahnfreundlich.html](https://www.zahnarztpraxis-berlin-treptow.de/1-212-516-Zahnmännchen-zur-Kariesprävention-ist-Siegel-der-Aktion-zahnfreundlich.html) | Germany | Berlin (city) | Private dental office, multiple dentists (one female, one male dentist) | DEGUZ | 2004 [51]^c^, 1999 |
| Kant&Co; Dres. Kant und Deinhardt | <https://www.zahnaerzte-kant.de/prophylaxe/prophylaxe-fuer-zu-hause> | Germany | Oldenburg (city) | Private dental office, multiple dentists (two female dentists) | DGZMK, DGKiZ, APW, BuKiZ, DGParo, DGSv, DGZH, GPZ, AG f. ZÄ Behindertenbehandlung^e^ | 1986, 2010 |
| Die Oldenburger Zahnärzte; Raßloff, Dech und Mikoleit | <https://die-oldenburger-zahnaerzte.de/leistungen/aesthetik/veneers/> | Germany | Oldenburg (city) | Private dental office, multiple dentists (six female, six male dentists) | DGZMK, DGÄZ, DGI, DGSZM, IAOMS, AOCMF, GA-OMS, ITI, DGZ, BDO | 1988, 2006, 2008, 1990, 1987, 2001, 2008, 2018, 2012, 2008, 2015, 2017 |
| Zahnarztpraxis Ottensen; ZA Meyer-Hamme | <https://www.implantologie-hamburg-ottensen.de/zahnmedizin-ueberempfindlichkeit-durch-saeureschaeden/> | Germany | Hamburg (city) | Private dental office, multiple dentists (two male dentists) | – | 2016 [52]^f^, N/A |
| Zahnärzte an der Kleinmachnower Schleuse; Prof. Naumann, Dr. Kiessling und Kollegen | <https://www.zahnarztkleinmachnow.de/zahnarzt/behandlung/behandlungsplanung.html> | Germany | Stahnsdorf (rural) | Private dental office, multiple dentists (two female, one male dentist) | DGZMK, DGI, DGParo, DGPro, DGFDT, APW, ITI, IADR, AAP, DGÄZ | 1998 [40]^c^, 1998 [41]^c^, 2012 [42]^c^ |
| Zahnarztpraxis Dr. Treuheit | <https://zahnarzt-treuheit.de/wp-content/uploads/2014/03/patienteninformation_vorsicht_saeure.pdf>  <http://www.zahnarzt-treuheit.de/wp-content/uploads/2014/03/patienteninformation_vorsicht_saeure.pdf> | Germany | Roßtal (rural) | Private dental office, multiple dentists (one female, one male dentist) | DGZMK, DGL, DGCZ | 1983 [53]^c^, 2016 [54]^g^ |
| Zahnarztpraxis Dr. Springer | <https://www.nordseezahnarzt.de/kopie-von-bleaching> | Germany | Husum (town) | Private dental office, multiple dentists (one female, one male dentist) | – | 2014, N/A |
| Dreiländer Zahnärzte; Dres. Ivanovas | https://www.zahnärzte-ivanovas.de/index.php?Patienten-Ratgeber-gesunde-Zaehne | Germany | Ravensburg (town) | Private dental office, multiple dentists (one female, one male dentist) | DGÄZ | 2005 [55]^c^, 2005 [56]^c^ |
| Zahnarztpraxis Dres. Münch | <https://www.drs-muench.de/spektrum/fuellungstherapie-in-nanotechnologie/> | Germany | Hirschaid (rural) | Private dental office, multiple dentists (one female, one male dentist) | DGZMK, DGI, LAGZ, APW, DGKiZ^e^ | 1980 [57]^c^, 2010 |
| Praxis Dr. Peintinger | <https://www.dr-peintinger.de/fuellungstherapie> | Germany | Traitsching (rural) | Private dental office, multiple dentists (one female, one male dentist) | – | 1995, 2017 |
| Zahnärzte Blasheim; Dr. Lückingsmeyer und ZÄ Halstenberg | <https://www.zahnaerzte-blasheim.de/information/ernaehrung-und-zahngesundheit-bei-kindern/>  <https://www.zahnaerzte-blasheim.de/schwerpunkte/ganzheitliche-ernaehrungsberatung/> | Germany | Lübbecke (town) | Private dental office, multiple dentists (one female, one male dentist) | – | 1988 [58]^c^, N/A |
| Zahnarzt Dr. Terhorst | <https://www.dr-terhorst.de/wissenswertes/die-zaehne-vor-saurem-schuetzen> | Germany | Dinslaken (town) | Private dental office, multiple dentists (two male dentists) | DGI, BDIZ | 1999, 2017 |
| Zahnärzte Saaland; ZA Jankowski | <https://www.zahnaerztesaarland.de/news/saeureschaeden-zahnschmelz-sodbrennen>  <https://www.zahnaerztesaarland.de/news> | Germany | Saarlouis (town) | Private dental office, multiple dentists (three female, two male dentists) | DGI, APW | 2009, 1977 [59]^c^, 2011, 2018, 2019 |
| Dres. Pierchalla und Kollegen | <https://www.dr-pierchalla.de/leistungen/zaehneknirschen-saeureschaeden/> | Germany | Münster (city) | Private dental office, multiple dentists (two male, one female dentist) | DGZMK, DGParo, DGI, APW | 1983 [60]^c^, 2003, 2011 |
| Die Zahnarztpraxis 24; Dres. Bernhardt und Mrowietz | <https://www.die-zahnarztpraxis24.de/saeureschaeden.html> | Germany | Berlin (city) | Private dental office, multiple dentists (three male dentists) | DGZMK, DGCZ, DGÄZ^e^ | 1982, 1991, 1986 |
| Zahnarztpraxis Dr. Wagner und Kollegen | [https://www.zahnarzt-dr-wagner.de/1-212-516-Zahnm%C3%A4nnchen-zur-Kariespr%C3%A4vention-ist-Siegel-der-Aktion-zahnfreundlich.html](https://www.zahnarzt-dr-wagner.de/1-212-516-Zahnmännchen-zur-Kariesprävention-ist-Siegel-der-Aktion-zahnfreundlich.html) | Germany | Balingen (town) | Private dental office, multiple dentists (two male dentists) | BDIZ | 1984, 2012 |
| Zahnärzte Dr. Köhler und Partner | <https://blog.zahnarzt-dr-koehler.de/gesunde-kinderzaehne-auch-in-der-weihnachtszeit/> | Germany | Pattensen (town) | Private dental office, multiple dentists (one female, one male dentist) | DGZMK, DGKiZ, APW, DGFDT, DGI, DGZS, DGParo^e^ | 2007, 2008 |
| Dr. Bartmann und Kollegen | <https://www.zahnarzt-minden.de/zahnarzt-lexikon/> | Germany | Minden (town) | Private dental office, multiple dentists (one female, one male dentist) | DGZMK^a^ | 1995, 2006 |
| Zahnarztpraxis Lenneps und Nagel | <https://www.lenneps-nagel.de/news/> | Germany | Berlin (city) | Private dental office, multiple dentists (one female, one male dentist) | – | 1980 [61]^f^, 1995 [62]^f^ |
| Praxisklinik Dr. Zastrow und Kollegen | <https://www.zahnarzt-dr-zastrow.de/aktuelles/karies-und-parodontitis-prophylaxe-das-koennen-sie-bei-der-taeglichen-zahnpflege-tun/> | Germany | Wiesloch (town) | Private dental office, multiple dentists (two female, two male dentists) | DGI | 2004, 2004, 2020 [63]^c^, N/A |
| Zahnarztpraxis Dres. Lotz | https://www.zahnärzte-lotz.de/ | Germany | Ahrensburg (town) | Private dental office, multiple dentists (one female, one male dentist) | – | 1978 [64]^c^, N/A |
| Zahnärzte Panorama1; Dr. Zahmann und ZÄ Jurela | <https://www.zahnarzt-praxis-berlin-mitte.de/zahnbleaching-in-berlin-schonende-aufhellung.html> | Germany | Berlin (city) | Private dental office, multiple dentists (one female, one male dentist) | DGZMK, DGCZ, DGI, DGÄZ, DGZ, DGZH | 2002, 2002 |
| Zahnarztpraxis Barzani | https://www.zahnärzte-hannover-langenhagen.de/gesundheitsnews/ | Germany | Langenhagen (town) | Private dental office, multiple dentists (one female, one male dentist) | – | 2004 [65]^g^, N/A |
| Zentrum für gesunde Zähne; Dr. Stoiber | <https://dr-stoiber.de/das-koennen-wir/dentalernaehrungsberatung/> | Germany | Dachau (town) | Private dental office, multiple dentists (one female, one male dentist) | BDIZ, DGI, GZM, DGET, LAGZ | 1990 [66]^c^, 2011 |
| Zahnarzt Dr. Böhme und Kollegen | <https://www.zahnarztpraxis-freiburg.de/de/E> | Germany | Friburgh (city) | Private dental office, multiple dentists (three female, one male dentist) | DGZMK, DGCZ^e^ | 1998 [67]^c^, N/A, N/A, N/A |
| Zahnarztpraxis an der Luftbrücke; Dr. Janssen | <https://www.zahnarzt-in-tempelhof.de/aktuelles/warum-zahnprophylaxe-fuer-vegetarier-besonders-wichtig-ist/> | Germany | Berlin (city) | Private dental office, multiple dentists (one female, one male dentist) | – | 1998 [68]^c^, N/A |
| Zahnärztliche Praxis Dres. Solaty und Becker | https://www.zahnarzt-hamburg-ärztehaus-volksdorf.de/gesundheitsnews/ | Germany | Hamburg (city) | Private dental office, multiple dentists (three female, two male dentists) | – | 1981 [69]^c^, 1984 [70]^c^, N/A, N/A, N/A |
| Zahnarztpraxis Dr. Meißner | <https://www.praxis-meissner-berlin.de/gesundheitsnews/> | Germany | Berlin (city) | Private dental office, multiple dentists (two female, one male dentist) | – | 1993, 2014, 1997 |
| Zahnarztpraxis Kleinschmidt | <https://www.zahnarztpraxis-kleinschmidt.de/zahnerosionen-zaehne-in-der-saeurefalle/> | Germany | Berlin (city) | Private dental office, multiple dentists (one female, one male dentist) | – | 1990, 2017 |
| Zahnärzte Drs. Roes | <https://www.zahnarztroes.de/zahnmedizinische-news/> | Germany | Bonn (city) | Private dental office, multiple dentists (one female, one male dentist) | – | 1988 [71]^f^, 1988 [72]^f^ |
| Zahnarztpraxis Dr. Nowak | <https://www.zahnarztnowak.de/zahnerhaltung/bulimie-essstoerung-zahnschaeden>  <https://www.zahnarztnowak.de/artikel/zahnschmelzschaeden-manager-besonders-betroffen> | Germany | Berlin (city) | Private dental office, multiple dentists (two male dentists) | DGZMK, DGI, DGZI | 1988 [73]^c^, N/A |
| Zahnarztpraxis Dres. Reinthaler und Hagn | <https://www.reinthaler-hagn.de/component/content/article?id=35:fuellungen-und-inlays> | Germany | Greding (town) | Private dental office, multiple dentists (five female, one male dentist) | DGZMK, DGI, DGOI, BDIZ, APW, DGZS^e^ | 1992, 2004, 2004, 2014, 2012, 2017 [74]^c^ |
| Praxis für Zahnheilunde; Dres. van der Straeten | <https://www.zahn-spezialisten-bonn.de/ratgeber-zaehne-erosionen/> | Germany | Bonn (city) | Private dental office, multiple dentists (one female, one male dentist) | DGZMK, DGI, DGParo, APW, DGFDT, DGPro^e^ | 1990, 1996 |
| Zahnarzt Berlin MITTE; Prof. Olze und Kollegen | <https://www.schoene-zaehne-berlin.de/4-quadrantensanierung-bisshebung/> | Germany | Berlin (city) | Private dental office, multiple dentists (three male, two female dentists) | DGZMK, DGÄZ | 1991, 1978, 2004, 2002, 2017 |
| Praxis Conrad48B; Dr. Riefenstahl | <https://www.riefenstahl-partner.de/gesundheitsnews/> | Germany | Berlin (city) | Private dental office, multiple dentists (one female, one male dentist) | – | 1992 [75]^c^, 2016 [76]^g^ |
| Zahnarzt Dres. Hüttemann | <https://www.zahnarzt-huettemann.de/aktuelles/ansicht/bisshebung-nach-dem-dahl-konzept.html> | Germany | Bühlertal (rural) | Private dental office, multiple dentists (two female, one male dentist) | – | 1997 [77]^g^, 2010 [78]^c^, N/A |
| Zahnarzt Lorke | <https://zahnarzt-lorke.de/leistungen/die-zweiten-zaehne/lachgassedierung-2/> | Germany | Würzburg (city) | Private dental office, multiple dentists (three female, one male dentist) | DGZMK, DGKiZ, DGÄZ^e^ | 2018 [79]^f^, N/A, N/A, N/A |
| Zahnärztliche Gemeinschaftspraxis Dr. Schlotmann | <https://www.schlotmann-zahnarzt.de/dentale-erosionen/> | Germany | Menden (town) | Private dental office, multiple dentists (two male dentists) | DGZMK, DGFDT, DGZS^e^ | 1990, N/A |
| Zahnarztpraxis am Kreuzberg; Dr. Eigenbrodt | <https://www.zahnarztpraxis-am-kreuzberg.de/aktuelles/neuigkeiten/so-stark-hilft-die-verduennung-von-fruchtsaeften-gegen-erosionen/>  <https://www.zahnarztpraxis-am-kreuzberg.de/aktuelles/neuigkeiten/detail/so-stark-hilft-die-verduennung-von-fruchtsaeften-gegen-erosionen> | Germany | Berlin (city) | Private dental office, multiple dentists (two male dentists) | DGZMK, DGFDT, DGParo, AG f. ZÄ Behindertenbehandlung^e^ | 1992, N/A |
| Lieblings-Zahnarzt; Dr. Steinbach | <https://www.lieblings-zahnarzt.de/diaet-fuer-die-zaehne/> | Germany | Cologne (city) | Private dental office, multiple dentists (five female, one male dentist) | – | 2008, 2018, 2018, 2016, 2017, 2018 |
| Dr. Tosse | <https://www.zahnarzt-in-rheinberg.de/cms/index.php/vorsorge/11-erosion-von-zahnschmelz> | Germany | Rheinberg-Orsoy (town) | Private dental office, multiple dentists (one female, one male dentist) | DGZMK, DGI, BDO, DGL, AALZ, AKOPOM, NAgP | 1995, 2006 [80]^c^ |
| Zahnarzt Dr. Gensler | [https://www.zahnarzt-gensler.de/f%C3%BCr-gesunde-z%C3%A4hne/zahnerosion/](https://www.zahnarzt-gensler.de/für-gesunde-zähne/zahnerosion/)  <https://www.zahnarzt-gensler.de/2012/04/06/was-ist-zahnerosion/> | Germany | Mellrichstadt (town) | Private dental office, multiple dentists (one female, one male dentist) | – | 1998 [81]^c^, 2004 [82]^c^ |
| Zahnarzt Tussing | <https://www.zahnarzt-giessen.de/glossar/E-Erosion.html> | Germany | Gießen (town) | Private dental office, multiple dentists (one female, one male dentist) | DGZMK, DGI, DGZS, DGZSM | 1997, 2011 |
| zahn:gesund; Dres. Deußen | <https://zahnaerzte-deussen.de/erosionen-welche-lebensmittel-und-medikamente-sind-gefaehrlich-fuer-die-zaehne/>  <https://zahnaerzte-deussen.de/tag/zahnarzt/> | Germany | Cologne (city) | Private dental office, multiple dentists (two female, one male dentist) | DGZMK, DGI, DGParo, IAA, AAID, AAE, IADT, APW, FVDZ, IAMM, GCI, DGKiZ, IAPD | 2002 [83]^c^, 2002, 2018 |
| Zahnklinik Dres. Jung | <https://zahnklinik-jung.de/zahnerosion-laesst-sich-wirksam-stoppen/> | Germany | Pfungstadt (town) | Private dental office, multiple dentists (five male, two female dentists) | DGZMK, DGI, BDIZ, DGCZ, DGÄZ, NAgP DGL, DÄGfA, DGCURAC, DRG, EADMFR, ESR, IADR, IADMFR, ISCAS, SGDMFR, RSNA | 1989 [84]^c^, 1988 [85]^c^, 1989, 1995, 2008, 2020 [86]^g^, N/A |
| Zahnarztpraxis Dres. Saupe und Schwarz | https://zahnärzte-seehof.de/zahnerosion-saeure-als-zahnfreind-nr-1/ | Germany | Teltow (town) | Private dental office, multiple dentists (two female, two male dentists) | – | 2011, 2011, 2014, 2018 |
| Zahnarztpraxis Leichsenring-Schlenz | <http://www.laecheln-syke.de/behandlungsspektrum_erosion.html> | Germany | Syke (town) | Private dental office, multiple dentists (two female dentists) | DGZMK, DGParo, DGÄZ, DG Pro^e^ | 1987, 2015 |
| Zahnarzt Zentrum Hannover; ZA Zaroban | <https://zahnarzt-zentrum-hannover.de/lexikon/erosion/> | Germany | Langenhagen (town) | Private dental office, multiple dentists (three male, two female dentists) | – | 2006, 1991, 2016, N/A, N/A |
| MunichDent; ZA Weiß | <https://www.munich-dent.de/unsere-leistungen/zahnerhalt-muenchen/zahnerosion-behandlung-in-muenchen/> | Germany | Munich (city) | Private dental office, multiple dentists (two female, one male dentist) | – | 2013 [87]^g^, 2015 [88]^c^, 2012 [89]^c^ |
| Gemeinschaftspraxis für Zahnheilkunde; Dres. Göbel und Neff | <https://www.zahnaerzte-goebel-neff.de/spektrum/prophylaxe/zahnerosion/> | Germany | Filderstadt (town) | Private dental office, multiple dentists (two male dentists) | DGZMK, APW, DGI, DGParo, DGZ, DGET, DGÄZ, BDIZ^e^ | 1989, 1989 |
| Zahnarztpraxis Ottensen; ZA Meyer-Hamme | <https://www.zahnarztpraxis-ottensen-hamburg.de/dentale-erosion-was-ist-es-und-wie-entsteht-sie/>  <https://www.zahnarztpraxis-ottensen-hamburg.de/zahnmedizin-dentale-erosionen-schaedigen-die-zahnoberflaeche/> | Germany | Hamburg (city) | Private dental office, multiple dentists (two male dentists) | – | 2016 [52]^f^, N/A |
| Zahnarztpraxis Dr. Wilde | <https://dr-imogen-wilde.de/erosion/> | Germany | Öhringen (town) | Private dental office, multiple dentists (four female, one male dentist) | DGZMK, AGZMB, AK PP | 1988 [90]^c^, 1982 [91]^g^, N/A, N/A, N/A |
| Zahnarztpraxis Dres. Schmidt und Beretz | <https://www.zahnarzt-wennigsen.com/dentale-erosionen/>  <https://www.zahnarzt-wennigsen.com/tag/dentale-erosionen/> | Germany | Wennigsen (rural) | Private dental office, multiple dentists (three male, one female dentist) | DGZMK, DGParo, DGNP | 1986, 2009, 1980, 1997 |
| PAN-Klinik; Dres. Eichhorn und Teymourtash | <https://www.pan-zahnheilkunde.de/zahn-glossar/erosion/> | Germany | Cologne (city) | Private dental office, multiple dentists (six female, one male dentist) | DGZMK, DGI, DGParo, APW, DGÄZ, DGET, AK Halitosis, IADT, DGKiZ, NAgP | 1994, 2001 [92]^c^, 2011, 2010, 2018, 2019, N/A |
| Zahnarztzentrum Bochum; Dr. Leugner und ZA Leugner | <https://zahnarzt-herne.blogspot.com/2013/01/zahnarzt-herne-news-erosion.html> | Germany | Bochum (city) | Private dental office, multiple dentists (two male, one female dentist) | DGZMK^e^ | 1987 [93]^c^, 1997, 2002 |
| Dr. Philipp Baumgarten und Dr. Kristina Baumgarten | <https://www.zahnaerzte-baumgarten.de/leistungen/behandlung-von-bioerosionen> | Germany | Siegen (city) | Private dental office, multiple dentists (two female, one male dentist) | DGI | 2000 [94]^c^, 2006 [95]^g^, N/A |
| Niliusklinik; Dres. Nilius | <https://www.niliusklinik.de/blog/gesundes-obst-zahnerosion-durch-fruchtsaeure/> | Germany | Dortmund (city) | Private dental office, multiple dentists (two female, one male dentist) | DGZI | 1999 [96]^c^, 1999 [97]^c^, N/A |
| Zahnhofen; Giannone und Kollegen | <https://www.zahnhofen.de/patienten-service/zahnlexikon/detail/erosion/> | Germany | Manheim (city) | Private dental office, multiple dentists (two male dentists) | – | 1990 [98]^c^, N/A |
| PearlDent; ZA Nisafi | <https://pearldent.de/zahnerosion/> | Germany | Berlin (city) | Private dental office, multiple dentists (one female, one male dentist) | DGZMK, DGParo, DGPro, APW, DGÄZ | 2011, N/A |
| Zahnmedizinisches Zentrum Aschaffenburg; Dr. Seifert und ZA Hijazi | <https://zmz-aschaffenburg.de/abrasionen-erosionen-und-deren-behandlung-in-funktion>  <https://zmz-aschaffenburg.de/> | Germany | Aschaffenburg (town) | Private dental office, multiple dentists (two male dentists) | – | 1997 [99]^c^, 2014 |
| Zahnarztpraxis Dr. Naser und Kollegen | <https://www.zahnarztpraxis-drnaser.de/gesundheitsnews/> | Germany | Möckmühl (town) | Private dental office, multiple dentists (three female, one male dentist) | – | 1982 [100]^c^, N/A, N/A, N/A |
| Zahnärzte Dres. Rust | <https://www.zahnaerzte-rust.de/gesundheitsnews/> | Germany | Bechtholsheim (rural) | Private dental office, multiple dentists (two male, one female dentist) | – | 2003 [101]^c^, 2003 [102]^g^, N/A |
| Zahnärztezentrum Hüttenberg; Dr. Krauhausen | <https://www.zahnaerztezentrum-huettenberg.de/neues-von-ihrem-zahnaerztezentrum-huettenberg-langgoens/>  (<https://www.zahnaerztezentrum-huettenberg.de/1859-sauer-ist-nicht-lustig-2/>)  (<https://www.zahnaerztezentrum-huettenberg.de/1852-zahngesund-durch-die-schwangerschaft/>) | Germany | Hüttenberg (rural) | Private dental office, multiple dentists (two male, one female dentist) | DGZMK, GZM, BdiZ, DGParo, DGFDT^e^ | 1986, 2014, N/A |
| Zahnärzte an der Au; Dres. Sohlich und Behrens-Bock | <https://www.zahnaerzte-an-der-au.de/alterszahnheilkunde.html> | Germany | Kiel (city) | Private dental office, multiple dentists (three female, two male dentists) | DGZMK, DGI, DGParo, DGKiZ, DGKFO, DGET, DGZ, APW^e^ | 1993, 2009, 2014, 2004, 2017 |
| Zahnarztpraxis Dres. Veit | <https://www.zahnarzt-dr-veit.de/Leistungen/schmerzen.html> | Germany | Munich (city) | Private dental office, multiple dentists (two female, one male dentist) | – | 2000, 1972, 2006 |
| Zahnarztzentrum am Potsdamer Platz; ZA Dubinskij | <https://www.zahnarzt-am-potsdamer-platz.de/prophylaxezentrum/hypersensibilitaet> | Germany | Berlin (city) | Private dental office, multiple dentists (two female, one male dentist) | – | N/A, N/A, N/A |
| Zahnärzte Dres. Bellmann | <https://zahnarzt-bellmann.de/prophylaxe/> | Germany | Langenhagen (town) | Private dental office, multiple dentists (one female, one male dentist) | DGZMK, DGI, BDIZ/EDI, DIZ, APW | 1998, 2005 [103]^c^ |
| Zahnarztpraxis Schönfeld | https://www.zahnarzt-gräfenroda.de/gesundheitsnews/ | Germany | Gräfenroda (rural) | Private dental office, multiple dentists (one female, one male dentist) | DGZMK, DGI, DGCZ, DGZI | 1989, 1989 |
| Zahnärzte Dres. Weigand und Brakebusch | <http://weigand-brakebusch.de/patienteninformationen/> | Germany | Hanover (city) | Private dental office, multiple dentists (one female, one male dentist) | DGZMK, DGI, DGParo, APW^e^ | 1993 [104]^c^, 2003 [105]^c^ |
| Zahnärzte Zentrum Ladeholz; Dres. Schwetje und Stichternath | <https://www.zahnarzt-sehnde.de/sportzahnmedizin> | Germany | Sehnde (town) | Private dental office, multiple dentists (four female, four male dentists) | DGzPRSport, IASD, DGOI | 1992, 2000, 2004, 2003, 2009, 2012, 2016, 2017 |
| Zahnarztpraxis Dres. Bettray | <https://www.dr-bettray.de/praxis/> | Germany | Kornwestheim (town) | Private dental office, multiple dentists (two male dentists) | – | 1969 [106]^c^, 1997 [107]^c^ |
| Zahnarztpraxis Spranke | <https://www.zahnarzt-spranke.de/2019/06/28/wichtige-zahnbehandlungen-waehrend-der-schwangerschaft/> | Germany | Dortmund (city) | Private dental office, multiple dentists (three female, three male dentists) | – | 2012, 2014, 2019, 2012, 2016, N/A |
| OXIDIO Zahnarztpraxis; Dr. Winkelmann | <https://www.oxidio.de/2016/08/19/erosionen-die-dritte-gefaehrliche-zahnkrankheit/> | Germany | Gärtringen (rural) | Private dental office, multiple dentists (three male dentists) | – | 1980 [108]^c^, 2019 [109]^f^, N/A |
| Zahnarztpraxis Dr. Kresic | <http://www.zahnarzt-kresic.de/index.php/therapie.html>  (<http://www.zahnarzt-kresic.de/index.php/aesthet-rekonstr-therapie.html>) | Germany | Hünstetten-Wallrabenstein (rural) | Private dental office, multiple dentists (one female, one male dentist) | DGZMK, DGI, DGParo, DGZ, APW^e^ | 1982, N/A |
| Zahnarztpraxis Dres. Croy | <https://www.zahnarzt-croy.de/praxisblog/vorsicht-sauren> | Germany | Zirndorf (town) | Private dental office, multiple dentists (two female, one male dentist) | DGZMK, DGÄZ, DGKFO, GMSCKFO, LAGZ, IGÄM, EAO, DGZSM | 1985 [110]^c^, 2014 [111]^f^, N/A |
| Praxis für Zahngesundheit; Dr. Goldhammer | <https://www.wagner-zahngesundheit.de/fullmouthrehabilitation> | Germany | Böblingen (town) | Private dental office, multiple dentists (two male dentists) | DGZMK, DGET, FVDZ | 2005, 1987 |
| Zahnarztpraxis Dr. Bauer | <http://zahn56.de/Information_Zahnersatz_Kosten_/Vermeidung_von_Zahnerkrankunge/Saureschaden/saureschaden.html> | Germany | Munich (city) | Private dental office, multiple dentists (two male dentists) | – | 1995 [112]^c^, N/A |
| Zahnarztpraxis Dr. Farspour | <https://www.zahnarzt-farspour.ch/bulimie/> | Foreign (Switzerland) | Pfäffikon (rural) | Private dental office, multiple dentists (two female dentists) | SSO | 1999, 2015 |
| Zahnarztpraxis Bubenberg; Dres. Mericske und Widmer | <https://www.zahnarztpraxis-bubenberg.ch/zahnfleisch-hygiene> | Foreign (Switzerland) | Berne (city) | Private dental office, multiple dentists (three male, two female dentists) | SSO, SGI, ZGKB, SVK, IAPD, SSOI, SSRD, SGZBB | 2001, 1992, 1976, 1982, N/A |
| Zahnmedizin im Schuerli; Dr. Jossen | <https://www.zahnmedizin-im-schuerli.ch/>  ([https://www.zahnmedizin-im-schuerli.ch/behandlungen#Erosionen+%28S%C3%A4uresch%C3%A4den+an+den+Z%C3%A4hnen%29](https://www.zahnmedizin-im-schuerli.ch/behandlungen" \l "Erosionen+(Säureschäden+an+den+Zähnen))) | Foreign (Switzerland) | Zofingen (town) | Private dental office, multiple dentists (two female dentists) | SSO, SSP | 2010, 2011 |
| Zahnärzte Dres. Heller, Kübler und Truninger | <https://www.zahnaerztezuerich.ch/index.php?id=277&L=0Dr>  <https://www.zahnaerztezuerich.ch/index.php?id=277&L=58>  <https://www.zahnaerztezuerich.ch/unser-behandlungsspektrum/zahnerosionen.html?L=0> | Foreign (Switzerland) | Zurich (city) | Private dental office, multiple dentists (four female, three male dentists) | SSO, SSO Zurich, SSP, ITI, AAP, SSRD, SGI-SSIO, SDI, SVK/ASP | 1981, 2002, 2003, 1990 [113]^c^, 2001, 1990 [114]^g^, 2010 |
| Zahnmedizin Luzern; Dr. Eppenberger | <https://zahnmedizinluzern.ch/aktuell/den-zaehnen-wird-zu-viel-saures-gegeben.html> | Foreign (Switzerland) | Lucerne (town) | Private dental office, multiple dentists (two female, two male dentists) | SSO | 1984, 1999, 2013, 2018 |
| Praxis Dr. Anding | <https://www.anding.ch/angebot.html> | Foreign (Switzerland) | Berne (city) | Private dental office, multiple dentists (two female dentists) | SSO | 1998, 2019 |
| Zahnarztpraxis Dr. Matthiessen | <https://www.zahnarzt-matthiessen.ch/index.php/zahnerkrankungen/zahnerosionen-und-abrasionen> | Foreign (Switzerland) | Mels (rural) | Private dental office, multiple dentists (three male dentists) | SSO | 2008 [115]^c^, 2008 [116]^g^, N/A |
| Centrella Zahnärzte; Dr. Centrella | <https://www.centrella.ch/de-ch/zahnarzt-praxis-oberrieden/kundenfeedback/erosionen-nach-essstorungen-bulimie.html> | Foreign (Switzerland) | Oberrieden (rural) | Private dental office, multiple dentists (two male dentists) | SSO^e^ | 1998, 2017 |
| Zahnarztpraxis Dres. Furini und Girardi | <https://www.furinilorenz.info/wer-darf-sie-behandeln/weitere-probleme/zahnerosionen/> | Foreign (Italy) | Gais (rural) | Private dental office, multiple dentists (two male dentists) | – | 1992 [117]^h^, 2012 [118]^h^ |
| Travel To Dentist; Punto Bianco multilimgual dental clinic | <https://traveltodentist.com/de/blog/probleme-und-zahnerkrankungen/die-abnutzung-die-erosion-dias-ausbrechen-die-abtragung-arten-von-zahnabrieb/> | Foreign (Moldova) | Chisinau (city) | Private dental office, multiple dentists (five male, one female dentist) | – | 2014 [119]^g^, 2016 [120]^g^, N/A, N/A, N/A, N/A |
| Zahnarzt-Praxis Prof. Dhom und Kollegen | <https://www.prof-dhom.de/aesthetische-zahnkorrekturen-mit-fuellmaterialien/> | Germany | Ludwigshafen (city) | Corporate dental office or private hospital group |  |  |
| Creative Zahnärzte MVZ GmbH | <https://www.creative-zahnaerzte.de/lexikon/erosion/> | Germany | Munich (city) | Corporate dental office or private hospital group |  |  |
| Zahnärzte Esplanade; MVZ Zahnärzte Esplanade GbR | <https://www.zahnarzthannover.eu/gesundes-essen-gute-zaehne-kauen-sie-sich-gesund/> | Germany | Hanover (city) | Corporate dental office or private hospital group |  |  |
| Zahnärzte Lemkamp Ammersee; Zahnarzt-Ammersee MVZ GmbH | <https://www.zahnarzt-ammersee.de/zahnerhalt> | Germany | Schondorf am Ammersee (rural) | Corporate dental office or private hospital group |  |  |
| LUX Zahnärzte; LUX ZAHNÄRZTE GmbH | <https://www.luxzahnaerzte.de/lexikon/erosion/> | Germany | Manheim (city) | Corporate dental office or private hospital group |  |  |
| Dr. Baader und Kollegen MVZ GmbH | <https://www.drbaader.de/presse/zahnmedizin-heute-zahnerosionen-aufloesung-des-zahnschmelzes-teil-iii/> | Germany | Mindelheim (town) | Corporate dental office or private hospital group |  |  |
| Viva Dental Düsseldorf; Viva Dental GmbH | <https://viva-dental.de/tag/erosion/>  <https://viva-dental.de/leistungsspektrum/zahnerosionen-grundsaetzlich-ohne-amalgam/>  <https://viva-dental.de/zahndefekte-durch-erosionen/>  <https://viva-dental.de/tag/erosionen/> | Germany | Dusseldorf (city) | Corporate dental office or private hospital group |  |  |
| Zahnärzte am Kurhaus; Zahnärzte am Kurhaus Wiesbaden MVZ | [https://www.zahnaerzte-am-kurhaus.de/eine-neue-h%c3%bclle-f%c3%bcr-den-zahn/keramikveneers](https://www.zahnaerzte-am-kurhaus.de/eine-neue-hülle-für-den-zahn/keramikveneers) | Germany | Wiesbaden (city) | Corporate dental office or private hospital group |  |  |
| Max 14; MVZ Max14 | <https://www.max14-zahnaerzte.de/restauration> | Germany | Munich (city) | Corporate dental office or private hospital group |  |  |
| Kinderzahnarzt Berlin; MVZ Kinder + Zahnarzt Berlin Dr. Hoberg Zahnmedizin GmbH | <https://www.kinderpluszahnarzt.berlin/kinderpluszahnarzt/erwachsene-zahnarzt/alterszahnmedizin> | Germany | Berlin (city) | Corporate dental office or private hospital group |  |  |
| AllDent; AllDent Zahnzentrum GmbH | <https://www.alldent-zahnzentrum.de/bulimie-zaehne.html> | Germany | Munich (city) | Corporate dental office or private hospital group |  |  |
| Zahnklinik Welldent; Zahnmedizinisches Versorgungszentrum Welldent GmbH | <https://www.welldent.de/infos-zahnklink-koeln/thema-zaehne-zahngesundheit/erosion> | Germany | Cologne (city) | Corporate dental office or private hospital group |  |  |
| Dentalklinik Frei; Dentalklinik Frei AG | <https://www.ihr-zahnarzt.ch/behandlung/zahnerosion> | Foreign (Switzerland) | Berne (city) | Corporate dental office or private hospital group |  |  |
| DZZ Lutz & Cantelmi AG; Dres. Lutz und Cantelmi | <https://www.dzz.ch/zahnmedizinisches-angebot.html> | Foreign (Switzerland) | Zofingen (town) | Corporate dental office or private hospital group |  |  |
| Praxis Nygren AG; Dr. Nygren | <http://www.praxis.nygren.ch/content/behandlungen/dentale-erosionen-saeureschaden/> | Foreign (Switzerland) | Herzogenbuchsee (rural) | Corporate dental office or private hospital group |  |  |
| Forrer Zahnärzte AG; Dres. Forrer | <https://forrerzahnaerzte.ch/download/Erosionen.pdf> | Foreign (Switzerland) | Weindelden (town) | Corporate dental office or private hospital group |  |  |
| swiss smile Schweiz AG | <https://www.swiss-smile.com/zahnmedizin/vorbeugen-und-zahngesundheit-erhalten/erosionsschaeden/> | Foreign (Switzerland) | Zurich (city) | Corporate dental office or private hospital group |  |  |
| zahnarztzentrum.ch AG | <https://zahnarztzentrum.ch/blog/2016/04/18/erosionen-was-tun/>  <https://zahnarztzentrum.ch/blog/tag/erosionen/> | Foreign (Switzerland) | Zurich (city) | Corporate dental office or private hospital group |  |  |
| Dentaland | <http://dentaland.info/de/taetigkeiten/aesthetische-zahnmedizin/erosion-der-zaehne/> | Foreign (Serbia) | Novi Sad (city) | Corporate dental office or private hospital group |  |  |
| Dentaprime; Dr. Miroslav Atanasov | <https://www.dentaprime.com/veneers> | Foreign (Bulgaria) | Varna (city) | Corporate dental office or private hospital group |  |  |
| Poliklinik für Präventive Zahnmedizin, Parodontologie und Kariologie; Universitätsmedizin Göttingen | <http://www.zahnerhaltung.med.uni-goettingen.de/leistungsspektrum>  (<http://www.zahnerhaltung.med.uni-goettingen.de/leistungsspektrum/prophylaxe/praevention-von-saeurebedingten-zahnschaeden-erosionen>)  (<http://www.zahnerhaltung.med.uni-goettingen.de/leistungsspektrum/minimalinvasive-restaurative-therapie/behandlung-saeurebedingter-zahnhartsubstanzdefekte-erosionen>) | Germany | Göttingen (city) | Public dental clinic or dental school |  |  |
| Klinik für Zahnerhaltungskunde und Parodontologie; Universitätsklinikum Freiburg | <https://www.uniklinik-freiburg.de/zahnerhaltung/team/oberaerzte/prof-dr-nadine-schlueter/erosionssprechstunde.html> | Germany | Friburgh (city) | Public dental clinic or dental school |  |  |
| Zahnärztliches MVZ; AmbulantesBehandlungsCentrum GmbH am Klinikum Nürnberg Süd | <https://www.klinikum-nuernberg.de/DE/ueber_uns/Fachabteilungen_KN/zentren/ABC/Dokumente_Bilder/Zahnarzt/Text_Fuellungstherapie.pdf> | Germany | Nuremberg (city) | Public dental clinic or dental school |  |  |
| Zahnerhaltungs- und präventive Zahnheilkunde; Universtitäsklinikum Giessen | <https://www.ukgm.de/ugm_2/deu/ugi_zpz/3888.html>  <https://www.ukgm.de/ugm_2/deu/ugi_zpz/4139.html> | Germany | Giessen (town) | Public dental clinic or dental school |  |  |
| Poliklinik für Konservierende Zahnheilkunde und Parodontologie; Universitätsklinikum Jena | <https://www.uniklinikum-jena.de/zzmk/kons-p-920.html> | Germany | Jena (city) | Public dental clinic or dental school |  |  |
| Klinik für Zahnerhaltung, Präventiv- und Kinderzahnmedizin; Universität Bern | <https://www.kons.zmk.unibe.ch/dienstleistungen/fuer_patienten/spezialsprechstunden/spezialsprechstunde_erosionen/index_ger.html> | Switzerland | Berne (city) | Public dental clinic or dental school |  |  |
| GZFA® GmbH (Gesellschaft für Zahngesundheit, Funktion und Ästhetik) | <https://www.gzfa.de/diagnostik-therapie/zahnaesthetik/erosion/>  <https://www.gzfa.de/aktuelles-wissen/news/detail/article/dentale-erosion-saeureangriff-auf-die-zaehne/> | Germany | Munich (city) | Dental society, dental regulatory body, public body, or insurance company |  |  |
| DGZMK (Deutsche Gesellschaft für Zahn-, Mund- und Kieferheilkunde) | <https://www.zahnmedizinische-patienteninformationen.de/documents/10157/1129556/268572_1594157_Was_sind_Erosionen.pdf> | Germany | Dusseldorf (city) | Dental society, dental regulatory body, public body, or insurance company |  |  |
| Aktion Zahnfreundlich e.V. | <https://www.zahnmaennchen.de/> | Germany | Berlin (city) | Dental society, dental regulatory body, public body, or insurance company |  |  |
| Initiative proDente e.V. | <https://www.prodente.de/zaehne/erkrankungen/empfindliche-zaehne.html> | Germany | Cologne (city) | Dental society, dental regulatory body, public body, or insurance company |  |  |
| AGZ-RNK (Arbeitsgemeinschaft Zahngesundheit für die Stadt Heidelberg und den Rhein-Neckar-Kreis) | <http://www.agz-rnk.de/agz/content/3/3_3/3_3_1/3_3_1_4/index.php>  <http://www.agz-rnk.de/agz/content/3/3_3/3_3_12/index.php>  <http://www.agz-rnk.de/agz/content/3/3_3/3_3_12/index.php?dspm=b> | Germany | Heidelberg (city) | Dental society, dental regulatory body, public body, or insurance company |  |  |
| Berufsverband der Kinder- und Jugendärzte e. V. | <https://www.kinderaerzte-im-netz.de/news-archiv/meldung/article/saeure-schadet-empfindlichen-kinderzaehnen/> | Germany | Cologne (city) | Dental society, dental regulatory body, public body, or insurance company |  |  |
| Landeszahnärztekammer Thüringen | <https://www.lzkth.de/lzkth2/cms_de.nsf/lzkth/zahnerosion.htm>  <https://www.lzkth.de/lzkth2/cms_de.nsf/($UNID)/F4451F81DEFC2E44C1257FF4003358E1?OpenDocument&NavDocID=185b648ba0566f3fc1257b80002f8eda&q=zahnerosion> | Germany | Erford (city) | Dental society, dental regulatory body, public body, or insurance company |  |  |
| Landeshauptstadt Stuttgart, Gesundheitsamt, Sachgebiet Zahngesundheit | <https://www.stuttgart.de/medien/ibs/web-1-x-1-fuer-kinderzaehne-2021.pdf> | Germany | Stuttgart (city) | Dental society, dental regulatory body, public body, or insurance company |  |  |
| BZÄK (Bundeszahnärztekammer) | <https://www.bzaek.de/fileadmin/PDFs/b/Milchzaehne_AzeV.pdf> | Germany | Berlin (city) | Dental society, dental regulatory body, public body, or insurance company |  |  |
| DFV (Deutsche Familienversicherung) | <https://www.deutsche-familienversicherung.de/zahnversicherungen/zahnzusatzversicherung/ratgeber/artikel/zahnschmelz-und-zahnschmelzabbau-ursachen-und-massnahmen-zur-vorbeugung/> | Germany | Frankfort-on-the-Main (city) | Dental society, dental regulatory body, public body, or insurance company |  |  |
| Zahnrat; Landeszahnärztekammern Brandenburg, Sachsen und Sachsen-Anhalt, Kassenzahnärztliche Vereinigung Sachsen-Anhalt und Landeszahnärztekammer Thüringen | <https://zahnrat.de/images/zahnrat-archiv/pdfs/ZahnRat-89.pdf> | Germany | Nieschütz (rural) | Dental society, dental regulatory body, public body, or insurance company |  |  |
| Stadt Hannover | <https://www.hannover.de/Leben-in-der-Region-Hannover/Gesundheit/Gesundheitsschutz/Kinder-und-Jugendgesundheit/Jugendzahnpflege/Zahnerosionen> | Germany | Hanover (city) | Dental society, dental regulatory body, public body, or insurance company |  |  |
| KZVH (Kassenzahnärztliche Vereinigung Hessen) | <https://www.kzvh.de/patienten/lexikonaz/KZVHO-LEX-EROSION.html> | Germany | Frankfort-on-the-Main (city) | Dental society, dental regulatory body, public body, or insurance company |  |  |
| TK (Techniker Krankenkasse) | <https://www.tk.de/techniker/gesundheit-und-medizin/praevention-und-frueherkennung/zahnvorsorge/fruechte-und-saefte-als-zahnkiller-2015640?tkcm=aaus> | Germany | Hamburg (city) | Dental society, dental regulatory body, public body, or insurance company |  |  |
| SSO (Schweizerische Zahnärzte-Gesellschaft) | <https://www.sso.ch/fileadmin/upload_sso/3_Patienten/3_Zahnerkrankungen/Zahnerosionen_d.pdf> | Foreign (Switzerland) | Berne (city) | Dental society, dental regulatory body, public body, or insurance company |  |  |
| ÖGK (Österreichische Gesundheitskasse) | <https://www.gesundheitskasse.at/cdscontent/?contentid=10007.847341&portal=oegksportal> | Foreign (Austria) | Saltzburgh (city) | Dental society, dental regulatory body, public body, or insurance company |  |  |
| Zahnjob; Kati Kliemann | <https://www.zahnjob.de/saeureschaeden.html> | Germany | Berlin (city) | Information service |  |  |
| DocMedicus Verlag GmbH & Co. KG | <http://www.zahngesundheit-online.com/Zahnerkrankungen/Dentale-Erosionen/> | Germany | Bad Münder (town) | Information service |  |  |
| Dr. Zahn; Joel Burghardt | <https://dr-zahn.de/zahnlexikon/abrasion/> | Germany | Mittenwald (rural) | Information service |  |  |
| Zahnwissen; Dr. Claus de Cassan | <https://www.zahnwissen.de/frameset_lexi.htm?lexikon_en-ez.htm> | Germany | Rickenbach (rural) | Information service |  |  |
| Deutsche Zahnarztauskunft | <http://www.deutsche-zahnarztauskunft.de/zahnaerzte/zahnarztnachrichten/singleview/?tx_ttnews%5Btt_news%5D=20122> | Germany | Gelsenkirchen (city) | Information service |  |  |
| OPTI-DENT GmbH | <https://www.zahnarzt-zahnbehandlung.com/index.php?option=com_content&view=article&id=71&Itemid=79&limitstart=19>  <https://www.zahnarzt-zahnbehandlung.com/index.php?option=com_content&view=article&id=235&Itemid=216&limitstart=38>  <https://www.zahnarzt-zahnbehandlung.com/index.php/component/content/article?id=235&start=37&Itemid=216>  <https://www.zahnarzt-zahnbehandlung.com/index.php/medizin-zahn/kariologie?showall=&start=37>  <https://www.zahnarzt-zahnbehandlung.com/index.php/mund-pflege/mund-hygiene?start=3> | Foreign (Switzerland) | Oberbüren (rural) | Information service |  |  |
| OPTI-DENT GmbH | <https://www.zahn-lexikon.com/index.php/z/380-zahnerosionen>  <https://www.zahn-lexikon.com/index.php/i/48-a-z/z-lexikon/380-zahnerosionen>  <https://www.zahn-lexikon.com/index.php/e/2871-erosion-der-zahn-hartsubstanz>  <https://www.zahn-lexikon.com/index.php/k/48-a-z/z-lexikon/380-zahnerosionen>  <https://www.zahn-lexikon.com/index.php/e/22-a-z/e-lexikon/2871-erosion-der-zahn-hartsubstanz>  <https://www.zahn-lexikon.com/e/28-joomla-promo/2871-erosion-der-zahn-hartsubstanz> | Foreign (Switzerland) | Oberbüren (rural) | Information service |  |  |
| OPTI-DENT GmbH | <https://opti-dent.ch/zahnlexikon/eintrag/erosion.html> | Foreign (Switzerland) | Oberbüren (rural) | Information service |  |  |

^a^Content provider’s location (rural or towns or cities with at least 100,000 inhabitants)

^b^Content provider (private dental office [single practitioner or multiple dentists], corporate dental office or private hospital group, public dental clinic or dental school, dental society or dental regulatory body or public body or insurance company, or information service).

^c^According to CV in published dissertation or master thesis.

^d^Not available.

^e^According to the member information of German Society of Dentistry and Oral Medicine (<https://www.zahnmedizinische-patienteninformationen.de/zahnarztsuche>) or Swiss Dental Association (<https://www.sso.ch/sso/zahnarztsuche.html>).

^f^According to public website.

^g^According to public profile at [https://www.linkedin.com](https://www.linkedin.com/), [https://www.xing.com](https://www.xing.com/), [https://www.facebook.com](https://www.facebook.com/), or <https://doktor.ch/>.

^h^According to directory of the professional representation.

| **Content provider’s name** | **URL** | **Country** | **Location^a^** | **Content provider^b^** | **Dental society membership** | **Year of examination** | **Upload date** | **Duration (minutes)** | **Number of likes** | **Number of dislikes** | **Number of comments** |
| --- | --- | --- | --- | --- | --- | --- | --- | --- | --- | --- | --- |
| 360°zahn | <https://www.youtube.com/watch?v=t4MrGyVxXFQ> | Germany |  | Corporate dental office or private hospital group |  |  | Dec 8, 2018 | 0.50 | 9 | 0 | 0 |
| Hirslanden-Gruppe | <https://www.youtube.com/watch?v=-QiP5aAX2BM> | Foreign (Switzerland) |  | Corporate dental office or private hospital group |  |  | Sep 1, 2015 | 6.17 | 12 | 1 | 0 |
| Zahnarzt Dr. Wajahat in Bad Kreuznach | <https://www.youtube.com/watch?v=ZMC6TAeeXmU> | Germany | Bad Kreuznach (town) | Private dental office, single practitioner (male) | – | 2014 [121]^c^ | May 2, 2020 | 10.75 | 21 | 0 | 0 |
| Dr. Frederike Fehrmann | <https://www.youtube.com/watch?v=ANppkjAgE98> | Germany | Heuchelheim (rural) | Private dental office, multiple dentists (five female dentist) | DGZMK, DGI, DGÄZ, DGCZ, DGL | 1999 [122]^c^, 1974 [123]^c^, N/A^d^, N/A, N/A | Jul 10, 2020 | 5.23 | 4 | 0 | 0 |
| Praxis für bewusste Zahngesundheit - Katsiotas & Hildebrandt - Zahnärzte | <https://www.youtube.com/watch?v=UEjWKae_eDg> | Germany | Burgdorf (town) | Private dental office, multiple dentists (one female, one male dentist) | DGZMK, DGÄZ, APW | 2008 [124]^e^, 2009 [125]^e^ | Feb 25, 2021 | 8.07 | 33 | 0 | 30 |
| Bayerischer Rundfunk | <https://www.youtube.com/watch?v=Jc1zKhIbjVo> | Germany |  | Information service |  |  | Jun 20, 2017 | 5.42 | 16 | 0 | 2 |
| MINI MED Studium | <https://www.youtube.com/watch?v=hHDk6Y01Zbw> | Foreign (Austria) |  | Information service |  |  | Dec 12, 2018 | 2.85 | 0 | 0 | 0 |

^a^Content provider’s location (rural or towns or cities with at least 100,000 inhabitants)

^b^Content provider (private dental office [single practitioner or multiple dentists], corporate dental office or private hospital group, public dental clinic or dental school, dental society or dental regulatory body or public body or insurance company, or information service).

^c^According to CV in published dissertation or master thesis.

^d^Not available.

^e^According to public website.

**Additional references from Multimedia Appendix 1**

1. Crass MWW. Zur Indikation enossaler Implantate im zahnlosen Unterkiefer. [Mainz]: Poliklinik für zahnärztliche Prothetik, Klinik und Polikliniken für Zahn-, Mund- und Kieferkrankheiten, Johannes Gutenberg Universität Mainz; 1989.

2. Plehwe I. Die Kraftabgabe des Doppeldelta-Loops bei Verwendung unterschiedlicher Loophöhen und Drahtqualitäten unter Berücksichtigung spezieller Schrifttumsquellen zu parodontalen Reaktionen auf orthodontische Kräfte. [Münster]: Poliklinik für Kieferorthopädie, Westphälische Wilhelms-Universität Münster; 1988.

3. Uhlig R. Zur Frage einer klinischen Bedeutung der Histaminfreisetzung beim Menschen. Untersuchungen an Mensch und Hund bei operativen Eingriffen und nach Applikation von Narkotika, Plasmasubstituten, Netzmitteln und Trypsin. [Marburg]: Abteilung für experimentelle Chirurgie und pathologische Biochemie, Chirurgische Universitätsklinik Marburg, Philipps-Universität Marburg; 1974.

4. Thiem-Müller G. Orientierende Untersuchung der Beratungssituation und -qualität in der Kieferorthopädischen Sprechstunde des Universitätsklinikums Hamburg-Eppendorf. [Hamburg]: Abteilung für Kieferorthopädie, Klinik für Zahn-, Mund- und Kieferkrankheiten, Universitätsklinikum Hamburg-Eppendorf, Universität Hamburg; 2003.

5. Grosch U. Die Bedeutung der Sexualhormone für den tubulären Transport von p-Aminohippursäure bei männlichen Ratten. [Jena]: Friedrich-Schiller-Universität Jena; 1992.

6. Steinhoff M. Amplifikation des erb B-2 Onkogenes als Prognosefaktor beim Mammakarzinom. [Münster]: Klinik und Poliklinik für Geburtshilfe und Frauenheilkunde, Westphälische Wilhelms-Universität Münster; 1997.

7. Malert J. Untersuchungen zum Dimensionsverhalten silikonhaltiger Abformmassen in Abhängigkeit von der Mundschleimhauttemperatur und der Raumtemperatur. [Göttingen]: Abteilung der Prothetik I, Zentrum Zahn-, Mund- und Kieferheilkunde, Fachbereich Medizin, Georg-August-Universität Göttingen; 1982.

8. Klabunde S. Zur Calcium-Steuerung der glattmuskulären Kontraktionskinetik. [Hamburg]: Abteilung für Vegetative Physiologie, Physiologische Institut, Universtitäts-Krankenhaus Eppendorf, Universität Hamburg; 1995.

9. Lessing R. Auswirkungen der Schraubendimension und des Gewindevorschnitts bei der Osteosynthese im Mittelgesicht - ein fluoreszenzoptischer Vergleich am Schaf. [Freiburg im Breisgau]: Klinik für Zahn-, Mund- und Kieferheilkunde, Abteilung Klinik und Poliklinik für Mund-, Kiefer- und Gesichtschirurgie, Albert-Ludwigs-Universität Freiburg im Breisgau; 1992.

10. Kettler H. Ergebnisse der chirurgischen Wurzelbehandlung mit dem CC-Cord-Besteck. [Hamburg]: Chirurgische Abteilung (Nordwestdeutsche Kieferklinik), Universitätsklinik und Poliklinik für Zahn-, Mund- und Kieferkrankheiten, Universitätskrankenhaus Hamburg-Eppendorf, Universität Hamburg; 1977.

11. Streletz E. Anthropometrische Messungen an Kindern im Alter von vier bis fünf Jahren in Wiesloch bei Heidelberg - Teilbereich Hautfettfalten -. [Frankfurt am Main]: Zentrum Kinderheilkunde, Klinikum der Johann Wolfgang Goethe-Universität Frankfurt am Main; 1986.

12. Seidel F. Die molekularen und zellulären Mechanismen der Quecksilberintoxikation. [Berlin]: Institut für Arbeitsmedizin, Sozialmedizin und Epidemiologie, Humblold-Universität Berlin; 1997.

13. Deisenhofer K-H. Untersuchungen von Wirksamkeit und Verträglichkeit einer muzinhaltigen Speichelersatzlösung in einer doppelblinde Cross-over Studie bei Patienten mit Xesostomie nach einer Radiatio im Kopf- und Halsbereich. [München]: Klinik für Hals-Nasen-Ohren Krankheiten des Zentralklinikums Augsburg, Ludwig-Maximilians-Universität München; 1993.

14. Weyland M. Elektrophoretische Untersuchungen der Speichelproteinadsorption (und initialen Biofilmbildung) auf Festkörperoberflächen in der Mundhöhle. [Homburg/Saar]: Klinik für Zahnerhaltung, Parodontologie und Präventive Zahnerheilkunde, Universitätsklinik des Saarlandes Homburg/Saar; 2009.

15. Tönissen EM. Web Archive. Available from: https://web.archive.org/web/20090316131446/http://www.nordseepraxis.de:80/teambilder.shtml [accessed Dec 13, 2021]

16. Spiekermann C. Facebook. Available from: https://www.facebook.com/chspiekermann [accessed Nov 29, 2021]

17. Diekmeier K. In-vivo-Korrosion am Praxispatienten, Nachweis von Palladium im Blut mittels laserangeregter Atomfluoreszenzspektrometrie (LAFS) - eine Pilotstudie -. [Berlin]: Zahnärztliche Prothetik II (Propädeutik), Klinik und Polikliniken für Zahn-, Mund- und Kieferheilkunde, Fachbereich Universitätsklinikum Benjamin Franklin, Freie Universität Berlin; 1994.

18. Stoltenow J. LinkedIn. Available from: https://www.linkedin.com/in/jörg-stoltenow-50a1b27a/ [accessed Nov 29, 2021]

19. Mönch (geb. Rottenberg) D. Die Patientenklientel der Poliklinik für Zahnerhaltung und Parodontologie der Freien Universität Berlin. [Berlin]: Abteilung für Zahnerhaltung und Parodontologie-Süd, Poliklinik für Zahnerhaltung und Parodontologie, Fachbereich für Zahn-, Mund- und Kieferheilkunde, Freie Universität Berlin;

20. Königer AJ. Vestibuläres Recruitment - Definition und Klinische Wertigkeit. [Würzburg]: Klinik und Poliklinik für Hals-, Nasen- und Ohrenkranke, Neurootologie, Julius-Maximilians-Universität Würzburg; 2006.

21. Dummler I. Mundgesundheit von Typ-I-Diabetikern. [Hamburg]: Abteilung für Zahnerhaltungskunde/Präventive Zahnheilkunde, Poliklinik für Zahn-, Mund- und Kieferkrankheiten, Universität Hamburg; 2000.

22. Borchard M. Isolierte Myozyten aus Meerschweinchenherzen als pharmakologisches Versuchsmodell. Elektrophysiologische Untersuchungen unter dem Einfluss verschiedener Ionen sowie extra- und intrazellulärer Applikation von g-Strophanthin. [Kiel]: Abteilung Pharmakologie, Zentrum Klinisch-Theoretisches Medizin II, Christian-Albrechts-Universität Kiel; 1987.

23. Brandt M. Entwicklung, Bau und Erprobung eines automatisierten pH-cycling-Systems. [Halle-Wittenberg]: Zentrum für Zahn-, Mund- und Kieferheilkunde, Poliklinik für Zahnerhaltung/Parodontologie, Martin-Luther-Universität Halle-Wittenberg; 1998.

24. Krettek S. Nachweis eines klonalen Geschehens in frühen Stadien von Mycosis fungoides. [Kiel]: Abteilung Dermatologie und Venerologie und Allergologie, Zentrum Konservative Medizin I, Christian-Albrechts-Universität Kiel; 1993.

25. Fremgen M. Vergleichende makroskopische und mikroskopische Untersuchungen zur Ausdehnung der Sekundärkaries. [Mainz]: Universitätsklinik und Poliklinik für Zahn-, Mund- und Kieferkrankheiten, Johannes-Gutenberg-Universität Mainz; 1986.

26. Riegger H. Die Wirkung von Chloropercha, CH-K-M, Großmann-Zement, Guttapercha, Jodoformpaste, Kloroperka und N2-normal auf den Nervusa mandibularis der Ratte. [Tübingen]: Abteilung für Kiefer- und Gesichtschirurgie, Zentrum für Zahn-, Mund- und Kieferheilkunde, Eberhard-Karls-Universität Tübingen; 1985.

27. Keiss R. Protektive Wirkung von Benetzungsmitteln auf humane Zellkulturen vor Austrocknung. [Greifswald]: Klinik und Poliklinik für Augenheilkunde, Institut für Anatomie und Zellbiologie, Ernst-Moritz-Arndt-Universität Greifswald; 2010.

28. Aeschbacher W. Die Häufigkeit von Zahnstellungsanomalien bei 9-jährigen Berner Schulkindern. [Bern]: Klinik für Kieferorthopädie, Universität Bern;

29. Meyer-Tsagolti TA. Die Entwicklung der Zahnmedizin in St. Petersburg von den Anfängen bis zur Gegenwart. [Zürich]: Medizinhistorisches Institut und Museum, Universität Zürich; 2006.

30. Faissler M. doktor.ch. Available from: https://www.doktor.ch/zahnarzt/faissler-manuel-adliswil-8134-zahnarzt.html [accessed Dec 13, 2021]

31. Bonatesta G. Die Entwicklung der Knochenplastik im Unterkiefer zwischen 1919 und 1939 in Deutschland. [Zürich]: Medizinhistorisches Institut und Museum, Universität Zürich; 1999.

32. Heiermann J. Web Archive. Available from: https://web.archive.org/web/20131028005323/http://quarree-dental.de/Heiermann.html [accessed Dec 13, 2021]

33. Hoischen T. Randspaltanalyse zwischen Vollkeramikkronen und Implantatpfosten des Procera-Systems. [Freiburg]: Universitätsklinik für Zahn-, Mund- und Kieferheilkunde, Poliklinik für Zahnärztliche Prothetik, Albert-Ludwigs-Universität Freiburg; 2008.

34. Untersuchungen zur Abgrenzung des Indikationsbereiches von Körperreinigungsmitteln. [Marburg]: Dermatologische Klinik und Poliklinik, Philipps-Universität Marburg; 1974.

35. Hasler RJ. Immunphänotypisierung von allogener und autofetaler Amnionmembran. [München]: Klinik und Poliklinik für Mund-, Kiefer und Gesichtschirurgie, Klinikum rechts der Isar, Technische Universität München; 2012.

36. Winterhalter D. Proportionalität euklidischer Abstände definierter extraoraler und intraoraler Punkte - Eine Machbarkeitsstudie. [Freiburg im Breisgau]: Department für Zahn-, Mund- und Kieferheilkunde, Klinik für Zahnärztliche Prothetik, Albert-Ludwigs-Universität Freiburg im Breisgau; 2015.

37. Runschke M. Zahnärzte am Lutherplatz. Available from: https://zahnaerzte-lutherplatz.de/#aerzte [accessed May 9, 2023]

38. Seibold (geb. Edler) M. Auswertung qualitativer Geruchsprofile bei pathologischer Geruchswahrnehmung unterschiedlicher Genese unter Berücksichtigung der Interaktion Geruch - Geschmack. [Würzburg]: Hals-, Nasen- und Ohren-Universitätsklinik und Klinik, Julius-Maximilians-Universität Würzburg; 1983.

39. Lang GJ. Dimensionstreue bei der computergestützten Bearbeitung dentaler Keramiken mit diamantierten Schleifkörpern unter Berücksichtigung ihres Verschleißverhaltens. [Frankfurt am Main]: Zentrum der Zahn-, Mund- und Kieferheilkunde, Poliklinik für Zahnerhaltungskunde, Johann Wolfgang Goethe-Universität Frankfurt am Main; 2002.

40. Kießling S. Klinisch-ätiologische Untersuchung zur Ursache von Lippen-Kiefer-Gaumenspalten sowie zum Vorkommen und zur Häufigkeit von Mikrosymptomen in Spaltträgerfamilien und in der Normalbevölkerung. [Berlin]: Klinik für Mund-, Kiefer- und Gesichtschirurgie, Medizinische Fakultät Charité, Humbold-Universität Berlin; 2001.

41. Naumann M. Restaurationsmöglichkeiten unkomplizierter Frontzahnfrakturen mit Dentinbeteiligung - eine vergleichende In-vitro-Untersuchung. [Berlin]: Abteilung für Zahnerhaltung, Zentrum Zahnmedizin, Medizinische Fakultät Charité, Humbold-Universität Berlin; 2001.

42. Dudka S. Hörrehabilitation durch Cochlea Implantation bei einseitiger Ertaubung. Einfluss auf Sprachverstehen, Lebensqualität, psychische Komorbiditäten und Tinnitusbelastung. [Berlin]: Klinik für Hals-, Nasen- und Ohrenheilkunde, Medizinische Fakultät Charité, Universitätsmedizin Berlin; 2018.

43. Basset U. Untersuchung des Gesichtsschädelaufbaus bei Patienten mit Arthropathie. [Freiburg im Breisgau]: Universitätsklinik für Zahn-, Mund- und Kieferheilkunde, Sektion für Zahnärztliches und Kieferchirurgisches Röntgen, Albert-Ludwigs-Universität Freiburg im Breisgau; 1993.

44. Ghahi BH. Langzeitergebnisse von Weichteilsarkom Resektionen der Extremitäten mit und ohne Gefäßersatz. [Berlin]: Zentrum für muskuloskeletale Chirurgie, Medizinische Fakultät Charité, Universitätsmedizin Berlin; 2020.

45. Lempa T. Präventive Gabe hämatopoietischer Wachstumsfaktoren beim elastaseinduzierten Lungenemphysem - Stereologische Untersuchungen an der Lewis-Ratte. [Hannover]: Institut für Funktionelle und Angewandte Anatomie, Zentrum Anatomie, Medizinische Hochschule Hannover; 2007.

46. Bernhardt K. Gewinnung und Charakterisierung von humenen Zementoblasten. [Göttingen]: Klinik für Zahnärztliche Prothetik, Zentrum Zahn-, Mund- und Kieferheilkunde, Medizinische Fakultät, Georg-August-Universität Göttingen; 2017.

47. Winkelmeyer CM. Analysis of tooth preparations for zirconia-based crowns and fixed dental protheses using stereolithography data sets. [Aachen]: RWTH Aachen;

48. Krauss M. Die Entstehung von Methanol im menschlichen Körper nach Aufnahme von Obst und Gemüse bei gleichzeitigem Ethanolgenuss. [Kiel]: Institut für Rechtsmedizin, Zentrum Klinisch-Theoretische Medizin II, Christian-Albrecht-Universität Kiel; 1998.

49. Hoffmann R. Untersuchungen der Toxizität dreier verschiedener ätherischer Öle auf die Expression der antimikrobiellen Peptide in der humanen Zellkultur. [Kiel]: Klinik für Mund-, Kiefer- und Gesichtschirurgie, Universitätsklinikum Schleswig-Holstein, Campus Kiel, Christian-Albrechts-Universität Kiel; 2006.

50. Imberg H. Vergleich von photometrischen Transmissions- und Reflexionseigenschaften dentaler Keramiken. [München]: Poliklinik für Zahnerhaltung und Parodontologie, Ludwig-Maximilians-Universität München; 2014.

51. Lau A. Laparoskopische Nierentumorchirurgie - Auswertung perioperativer Daten, Komplikationen und Follow-up von 300 Patienten der Klinik für Urologie an der Charité Campus Mitte. [Berlin]: Klinik für Urologie, Medizinische Fakultät Charité, Universitätsmedizin Berlin; 2011.

52. Ungoreit E. Tomorrowdent. Available from: https://tomorrow-dent.de/ueber-uns/ [accessed Jan 11, 2022]

53. Treuheit K-D. Traumatische Durchtrennung von Fingerstreckensehnen (Jahrgang 1977/78). [Erlangen-Nürnberg]: Chirurgische Klinik mit Poliklinik, Friedrich-Alexander-Universität Erlangen-Nürnberg; 1984.

54. Schuster V. Facebook. Available from: https://www.facebook.com/verena.hoeng/ [accessed Nov 29, 2021]

55. Ivanovas NS. Verätzungen des oberen Gastrointestinaltraktes. Ursachen - Diagnostik - Therapie. Auswertung eines 10 Jahres Zeitraums. [Köln]: Zentrum für Operative Medizin, Klinik und Poliklinik für Allgemein-, Viszeral- und Tumorchirurgie, Universität Köln; 2009.

56. Ivanovas S. Reparatur siloranbasierter Dentalkomposite. [München]: Poliklinik für Zahnerhaltung und Parodontologie, Ludwig-Maximilians-Universität München; 2011.

57. Münch W. Das Nasopharynxkarzinom: Äthiologie, Diagnostik und Therapie. Aus dem Krankengut der Jahre 1968 bis 1978 der Universitäts-Hals-Nasen-Ohren-Klinik Erlangen. [Erlangen-Nürnberg]: Klinik und Poliklinik für Hals-, Nasen- und Ohrenkranke, Friedrich-Alexander-Universität Erlangen-Nürnberg;

58. Lückingsmeyer E-G. Untersuchungen zur Anwendbarkeit dentaler Altersbestimmungsmethoden in der Anthropologie. [Giessen]: Justus-Liebig-Universität Giessen; 1990.

59. Bodtländer F-J. Mikroangiographische Untersuchungen druckstabilisierter Beckenkammtransplantate - Tierexperimentelle Untersuchungen -. [Mainz]: Klinik und Poliklinik für Zahn-, Mund- und Kieferkrankheiten, Kieferchirurgische Klinik, Johannes Gutenberg-Universität Mainz; 1978.

60. Pierchalla J. Die präoperative Aufklärung aus der Sicht eines Patienten : Eine Umfrage. [Berlin]: Abteilung für Allgemein-, Gefäß- und Thoraxchirurgie, Chirurgische Klinik und Poliklinik Universitätsklinikum Steglitz, Freie Universität Berlin;

61. Lenneps H. Web Archive. Available from: https://web.archive.org/web/20150526024752/http://lenneps-nagel.de/zahnaerzte.htm [accessed Dec 13, 2021]

62. Nagel J. Web Archive. Available from: https://web.archive.org/web/20150526024752/http://lenneps-nagel.de/zahnaerzte.htm [accessed Dec 13, 2021]

63. Krause SC. Einfluss verschiedener kieferorthopädischer Brackets auf die diagnostische Qualität in der 1,5 Testa Magnetresonanztomografie. [Homburg/Saar]: Klinik für Kieferorthopädie, Universitätsklinik des Saarlandes Homburg/Saar; 2021.

64. Lotz G. Die Zahnüberzahl im Oberkiefer-Frontzahngebiet unter besonderer Berücksichtigung der Odontoide. [Marburg]: Medizinisches Zentrum für Zahn-, Mund- und Kieferheilkunde, Philipps-Universität Marburg; 1982.

65. Barzani S. Facebook. Available from: https://www.facebook.com/shler.barzani.1 [accessed Nov 29, 2021]

66. Stoiber J. Spektralphotometrische Farbortbestimmung der weißen und grauen Hirnsubstanz. [München]: Institut für Rechtsmedizin, Ludwig-Maximilians-Universität München; 1990.

67. Böhme G. Festigkeitsprüfungen an verschiedenen Dentalkeramiken. [Freiburg im Breisgau]: Universitätsklinik für Zahn-, Mund- und Kieferheilkunde, Forschungsbereich Experimentelle Zahnheilkunde, Albert-Ludwig-Universität Freiburg im Breisgau; 1999.

68. Janssen U. Vergleichende In-Vitro-Untersuchung zum Bruchlastverhalten der Wurzelstiftsysteme Luscent Anchor® und Perma-Tex®. [Göttingen]: Abteilung Zahnerhaltung, Präventive Zahnheilkunde und Parodontologie, Zentrum Zahn-, Mund- und Kieferheilkunde, Georg-August-Universität Göttingen; 2001.

69. Becker H. Über den Einfluß von Vasokonstringentienzusatz und Probanolol-Prämedikation auf Lokalanästhetika-Krämpfe bei weissen Mäusen. [Hamburg]: Pharmakologisches Institut, Universität Hamburg; 1984.

70. Schwartz G. Das Plattenepithelkarzinom der Lippen. Retrospektive Studie der Heilungsergebnisse und Überlebensraten unter Berücksichtigung funktioneller und ästhetischer Aspekte der operativen Therapie aus den Jahren 1981 - 1991. [Würzburg]: Klinik und Poliklinik für Mund-, Kiefer- und Gesichtschirurgie, Julius-Maximilians-Universität Würzburg; 1991.

71. Roes A. Web Archive. Available from: https://web.archive.org/web/20101126133238/http://www.zahnarztroes.de:80/ [accessed Dec 13, 2021]

72. Roes-Tan R. Web Archive. Available from: https://web.archive.org/web/20101126133238/http://www.zahnarztroes.de:80/ [accessed Dec 13, 2021]

73. Nowak M. Idiopathisches Zungenbrennen - Untersuchungen zur Symptomatik und zum langzeitigen Verlauf. [Berlin]: Abteilung für Zentrale Aufnahme, Mundkrankheiten und Röntgenologie, Klinik und Poliklinik für Mund-, Kiefer- und Gesichtschirurgie, Fachbereich Zahn-, Mund- und Kieferheilkunde, Freie Universität Berlin;

74. Rist SM. Die Wirkung von Ghrelin auf die schlafassoziierte Regulation metabolischer Abläufe. [Tübingen]: Institut für Medizinische Psychologie, Eberhard Karls Universität Tübingen; 2020.

75. Riefenstahl PKO. Klinische Evaluation einer modifizierten Vestibulumplastik. [Freiburg im Breisgau]: Department für Zahn-, Mund- und Kieferheilkunde, Klinik für Mund-, Kiefer- und Gesichtschirurgie, Albert-Ludwigs-Universität Freiburg im Breisgau; 2017.

76. Kashua D. Facebook. Available from: https://www.facebook.com/photo.php?fbid=1115254368535641 [accessed Jan 11, 2022]

77. Hüttemann H. LinkedIn. Available from: https://www.linkedin.com/in/holger-hüttemann-4b43616b/ [accessed Nov 29, 2021]

78. Kist S. Pilotstudie. Der Effekt von sofortigem und verzögertem automatischen Haltungsfeedback auf das Wohlbefinden und die Haltung bei Personen mit sitzender Tätigkeit. [Tübingen]: Universitätsklinik für Zahn-, Mund- und Kieferheilkunde Tübingen, Abteilung Poliklinik für Zahnärztliche Prothetik und Propädeutik; 2013.

79. Wirthensohn (geb. Bohmann) L. Dr Moroni. Available from: https://dr-moroni.de/praxis/team/laura-wirthensohn/ [accessed May 9, 2023]

80. Böhm A. Untersuchung des periimplantären Gewebes mittels CT-Diagnostik - Experimentelle Studie. [Köln]: Zentrum für Zahn-, Mund- und Kieferheilkunde, Interdisziplinäre Poliklinik für Orale Chirurgie und Implantologie, Universität Köln; 2008.

81. Gensler J. Klinische Untersuchung zur dreidimensionalen Kephalometrie mit dem DigiGraph. [Erlangen-Nürnberg]: Poliklinik für Kieferorthopädie, Friedrich-Alexander-Universität Erlangen-Nürnberg; 2001.

82. Kemper AEM. Experimente mit dem Audiometer visible speech (Madsen) an verschiedenen HdO Hörgeräten, Vergleich verschiedener HdO Hörgeräte. [Würzburg]: Klinik und Poliklinik für Hals-, Nasen- und Ohrenkranke, Julius-Maximilians-Universität Würzburg; 2005.

83. Deußen D. Letalitätsanalyse pädiatrischer Schädel-Hirnverletzungen. [Frankfurt am Main]: Zentrum der Kinderheilkunde, Abteilung Pädiatrische Neurologie, Johann Wolfgang Goethe-Universität Frankfurt am Main; 2005.

84. Jung AR. Tierexperimentelle Untersuchung zur Biokompatibilität von teilstabilisiertem Zirkoniumoxid. [Mainz]: Klinik für Mund-, Kiefer- und Gesichtschirurgie, Johannes Gutenberg-Universität Mainz; 1988.

85. Jung T. Zur Abhängigkeit des Kaumusters von der Beschaffenheit des Nahrungssubstrates. [Düsseldorf]: Abteilung für Zahnärztliche Prothetik, Westdeutsche Kieferklinik Düsseldorf, Universität Düsseldorf; 1992.

86. S. J. LinkedIn. Available from: https://www.linkedin.com/in/jülide-s-276a82b8/ [accessed Nov 29, 2021]

87. Weiß MJ. LinkedIn. Available from: https://www.linkedin.com/in/michael-javier-weiß-a0865021/ [accessed Nov 29, 2021]

88. De Boer L. Die Häufigkeit odontogener Entzündungsbefunde bei Patienten vor und nach Herzklappenersatz. [Freiburg im Breisgau]: Department für Zahn-, Mund- und Kieferheilkunde, Klinik für Mund-, Kiefer- und Gesichtschirurgie, Albert-Ludwigs-Universität Freiburg im Breisgau; 2016.

89. Kyas D. Einfluss der medikamenten-induzierten Schlafendoskopie auf Therapieentscheidungen beim obstruktiven Schlafapnoe-Syndrom im Erwachsenen- und Kindesalter. [Leipzig]: Klinik und Poliklinik für Hals-, Nasen- und Ohrenheilkunde, Medizinische Falkultät, Universität Leipzig; 2017.

90. Wilde I. Rasterelektronenmikroskopische Untersuchungen an Dentikeln. [Tübingen]: Zentrum für Zahn-, Mund- und Kieferheilkunde, Poliklinik für Zahnerhaltung, Eberhard-Karls-Universität Tübingen; 1991.

91. Gerlach H. LinkedIn. Available from: https://www.linkedin.com/in/heinke-gerlach-b162796a/ [accessed Nov 29, 2021]

92. Trennheuser K. Wirksamkeit und Verträglichkeit von Mometasonfuroat-Nasenspray zur Therapie der chronischen Sinusitis. [Köln]: Institut für Medizinische Statistik, Informatik und Epidemiologie, Universität Köln; 2005.

93. Leugner GF. Die Effektivität der Stufenaufklärung bei ambulant kieferchirurgischen Eingriffen. [Bochum]: Knappschaftskrankenhaus Rechkinghausen, Ruhr-Universität Bochum; 1992.

94. Baumgarten P. Langzeit-Verlaufsbetrachtung bei Patienten nach Schrittmacherimplantation. [Freiburg im Breisgau]: Abteilung Innere Medizin III - Kardiologie, Medizinische Universitätsklinik und Poliklinik, Albert-Ludwigs-Universität Freiburg im Breisgau; 2001.

95. Baumgarten K. LinkedIn. Available from: https://www.linkedin.com/in/kristina-baumgarten-aa40a546/ [accessed Nov 29, 2021]

96. Nilius M. Untersuchungen zur Bindung von Arsen an Proteine des Hirns vom Meerschweinchen und Neuroblastom-Zellen der Maus in Gegenwart von Antidota mit vizinalen SH-Gruppen. [München]: Walther-Staub-Institut für Pharmakologie und Toxikologie, Ludwig-Maximilians-Universität München; 2001.

97. Nilius MO. Motivation zum Studium der Zahnmedizin. Eine Befragung von Studierenden an der Martin-Luther-Universität Halle-Wittenberg. [Halle-Wittenberg]: Universitätspoliklinik für Zahnärztliche Prothetik, Sektion Zahnärztliche Propädeutik, Martin-Luther-Universität Halle-Wittenberg; 2007.

98. Haffele M. Wirkung von prä- und interpartaler Antibiotikagabe bei nachgewiesenem Streptokokken-B-Antigen bei Schwangeren auf Geburstverlauf und-modus sowie auf das fetal outcome. [Heidelberg]: Universitäts Frauenklinik Heildelberg, Allgemeine Geburtshilfe und Frauenheilkunde mit Poliklinik, Ruprecht-Karls-Universität Heidelberg; 1992.

99. Seifert T. Primärstabilität dentaler Implantate - Einfluß von Art und Durchmesser der Implantate sowie Art der für die Untersuchung benutzter Knochenmaterialien. [Berlin]: Klinik für Zahn-, Mund- und Kieferheilkunde, Medizinische Fakultät der Charité, Universitätsmedizin Berlin;

100. Naser T. Transplantation von immunkompetenten Zellen beim schweren kombinierten Immundefekt SCID und beim Di-George-Syndrom -Alternativen zur Knochenmarktransplantation-. [Tübingen]: Abteilung für Pädiatrische Hämatologie der Kinderklinik, Eberhard-Karls-Universität Tübringen; 1983.

101. Rust G. Lernerfahrungen, Angstdisposition, Angstsensitivität und negative Erwartungen: Zur Vorhersage der Angst bei zahnärztlicher Behandlung. [Mainz]: Poliklinik für Kieferorthopädie, Johannes-Gutenberg-Universität Mainz; 2004.

102. Rust J. Xing. Available from: https://www.xing.com/profile/Julia_Rust [accessed Nov 29, 2021]

103. Bellmann M. Internetportale für die Vermittlung zahnmedizinischer Leistungen - Motivationen, Nutzen und Tauglichkeit aus Patientensicht. [Hannover]: Institut für Epidemiologie, Sozialforschung und Gesundheitssystemforschung, Medizinische Hochschule Hannover; 2009.

104. Weigand S. Das bakterielle Kontaminationsrisiko autologer Blutkonserven. Untersuchung des bakteriellen Kontaminationsrisikos in Bezug auf das verwendete Blutentnahmesystem. [Hannover]: Zentrale Anästhesieabteilung des orthopädischen Fachkrankenhauses Annastift Hannover, Medizinische Hochschule Hannover; 1996.

105. Rakebusch (geb. Asmussen) R. Erosionsgenese und prophylaktische Maßnahmen bei Patienten mit bulimischen Essstörungen. [Hannover]: Zentrum für öffentliche Gesundheitspflege, Abteilung Medizinische Psychologie, Medizinische Hochschule Hannover; 2005.

106. Bettray T. Quantitativ-immunologische Serumproteinbestimmungen nach Gelfiltration bei Insulinallergie einer Nichtdiabetikerin. [Tübingen]: Fachbereich Klinische Medizin, Medizinische Poliklinik, Eberhard-Karls-Universität Tübingen; 1971.

107. Bettray A. Stumpfes Bauchtrauma im Kindesalter. Eine retrospektive Untersuchung von 95 Patienten mit einem stumpfen Bauchtrauma von 1988 - 1997. [Freiburg im Breisgau]: Chirurgische Universitätsklinik, Abteilung Allgemein-Chirurgie und Viszeral-Chirurgie mit Poliklinik, Albert-Ludwigs-Universität Freiburg im Breisgau; 2000.

108. Winkelmann R. Kalziumkonzentrationsänderungen während der Blutglucoseregulierung mit dem Artificial Endocrine Pancreas - AEP. [Freiburg im Breisgau]: Chirurgische Universitätsklinik, Albert-Ludwigs-Universität Freiburg im Breisgau; 1980.

109. Winkelmann L. Oxidio. Available from: https://www.oxidio.de/wp-content/uploads/2020/10/Winkelmann_Interview_df_1_20.pdf [accessed Jan 20, 2022]

110. Croy P. Bekanntheitsgrad und Akzeptanz der Kieferorthopädischen Behandlung Erwachsener (Ergebnisse einer Fragebogenstudie). [Krems]: Department für Interdisziplinäre Zahnmedizin und Technologie, Donau-Universität Krems, Österreich; 2009.

111. Croy B. Semmelweis Universität. Available from: https://semmelweis.hu/oktatasi-centrum/files/2018/09/szakdolgozatok-2018jul13-1.pdf [accessed Jan 11, 2022]

112. Bauer MW. Optimierung des Dentinverbundes von Kompomerfüllungen in gemischten Klasse-V-Kavitäten. Eine in-vitro Studie. [München]: Poliklinik für Zahnerhaltung und Parodontologie, Ludwig-Maximilians-Universität München; 1998.

113. Schrader C. Farb- und Densitometrische Veränderungen natürlicher Zähne durch licht- und thermokatalytisches Bleichen. [Basel]: Zahnärztliches Institut, Abteilung für zahnärztliche Technologie, Werkstoffkunde und Propädeutik, Universität Basel; 1991.

114. Inglezos E. doktor.ch. Available from: https://www.doktor.ch/zahnarzt/inglezos-emmanouil-zuerich-8001-zahnarzt.html [accessed Dec 13, 2021]

115. Matthiessen U. Bedeutung der dreidimensionalen Darstellung der Nerv-Wurzelbeziehung bei Entfernung der dritten Molaren im Unterkiefer. [Zürich]: Zentrum für Zahn-, Mund- und Kieferheilkunde, Klinik für Zahn-, Mund- und Kieferkrankheiten und Kiefer- und Gesichtschirurgie, Universität Zürich; 2009.

116. Bandelier C. LinkedIn. Available from: https://www.linkedin.com/in/dr-christian-bandelier-088b99b3/ [accessed Nov 29, 2021]

117. Furini L. FNOMCeO. Available from: https://portale.fnomceo.it/cerca-prof/elenco.php [accessed May 23, 2023]

118. Girardi M. FNOMCeO. Available from: https://portale.fnomceo.it/cerca-prof/elenco.php [accessed May 23, 2023]

119. Vitalie O. LinkedIn. Available from: https://www.linkedin.com/in/osman-vitalie-92957b144/ [accessed Nov 29, 2021]

120. Strungaru A. LinkedIn. Available from: https://www.linkedin.com/in/anastasia-strungaru-444b61b3/ [accessed Nov 29, 2021]

121. Wajahat A. Sensitivierung von kolorektalen Karzinomzellen gegenüber Topoisomerase I inhibierende Zytostatika durch Einsatz von SMAC-Mimetika. [Mainz]: Institur für Toxikologie, Universitätsmedizin, Johannes-Gutenberg-Universität Mainz; 2018.

122. Fehrmann (geb. Laun) A-F. Patientencompliance bei marginaler Parodontitis. [Gießen]: Justus Liebig-Universität Giessen; 2001.

123. Laun (geb. Ludewig) U. In vitro Versuche über die kariesprophylaktische Wirkung eines Versiegelungsmaterials an Praemolaren und Molaren des Menschen. [Gießen]: Medizinisches Zentrum für Zahn-, Mund- und Kieferkrankheiten, Klinikum der Justus Liebig-Universität Gießen; 1976.

124. Katsiotas P. Praxis für bewusste Zahngesundheit. Available from: https://www.zahnbewusst.de/das-team/periklis-katsiotas/ [accessed May 28, 2023]

125. Hildebrandt J. Praxis für bewusste Zahngesundheit. Available from: https://www.zahnbewusst.de/das-team/jenny-hildebrandt/ [accessed May 28, 2023]
