# Supplementary material for: Quality of Patient-Centered eHealth Information on Erosive Tooth Wear: Systematic Search and Evaluation of Websites and YouTube Videos
Source: J Med Internet Res. 2024 Jan 31;26:e49514. doi: 10.2196/49514 (PMC10867746; doi:10.2196/49514)
Supplement: Multimedia Appendix 2 [file jmir_v26i1e49514_app2.doc]

**Multimedia Appendix 2:** Subdomains regarding technical and functional aspects (domain 1) were assessed using the LIDA instrument (version 1.2; Minervation) [37]. Ordinal scores of 0 (never or no), 1 (sometimes or partially), 2 (mostly), or 3 (always or yes) were given.

| Subdomain | Item | Median (IQR) | Range |
| --- | --- | --- | --- |
| **1.1 Accessibility** | | | |
|  | Does it work on a range of browsers?^a^ | 3 (3-3) | 3-3 |
|  | Is the information available full text without registration, log-in or subscription? | 3 (3-3) | 3-3 |
| **1.2 Usability** | | | |
|  | Is there a clear statement of who this website is for? | 2 (2-3) | 0-3 |
|  | Is the level of detail appropriate to their^b^ level of knowledge? | 3 (2-3) | 1-3 |
|  | Is the layout of the main block of information clear and readable? | 2 (2-3) | 0-3 |
|  | Is the navigation clear and well structured? | 2 (1-3) | 0-3) |
|  | Can you always tell your current location in the site? | 2 (1-3) | 0-3 |
|  | Is the colour scheme appropriate and engaging? | 3 (2-3) | 0-3 |
|  | Is the same page layout used throughout the site? | 3 (3-3) | 2-3 |
|  | Do navigational links have a consistent function?^c^ | 2 (2-2) | 0-3 |
|  | Is the site structure (categories or organisation of pages) applied consistently? | 3 (3-3) | 0-3 |
|  | Does it have an effective search function?^d^ | 0 (0-1) | 0-2 |
|  | Does the site provide effective browsing facilities? | 3 (2-3) | 0-3 |
|  | Does the design minimize the cognitive overhead? | 2 (2-3) | 0-3 |
|  | Does the site support the normal browser navigational tools? | 3 (3-3) | 2-3 |
|  | Can you use the site without third party plugins? | 3 (2-3) | 1-3 |
|  | Can the user make an effective judgment of whether the site applies to them? | 3 (2-3) | 0-3 |
|  | Is the website interactive? | 1 (0-2) | 0-3 |
|  | Can the user personalise their experience of using the site? | 0 (0-0) | 0-3 |
|  | Does the website integrate nontextual media? | 1 (1-2) | 0-3 |
| **1.3 Reliability** | | | |
|  | Does the site respond to recent events? | 1 (0-2) | 0-3 |
|  | Can users submit comments on specific content? | 0 (0-0) | 0-3 |
|  | Is site content updated at an appropriate interval? | 1 (0-2.5) | 0-3 |
|  | Is it clear who runs the site? | 3 (3-3) | 1-3 |
|  | Is it clear who pays for the site? | 3 (3-3) | 1-3 |
|  | Is there a declaration of the objectives of the people who run the site? | 2 (2-2) | 0-3 |
|  | Does the site report a clear content production method? | 1 (1-1) | 0-3 |
|  | Is this a robust method? | 0 (0-0) | 0-3 |
|  | Can the information be checked from original sources? | 0 (0-0) | 0-3 |

^a^Google Chrome version 109.0.5414.120, Firefox version 108.0.1, Microsoft Edge version 109.0.1518.78, and Apple Safari version 16.3 were tested.

^b^The patients.

^c^Websites were screened for broken links using a free online tool (https://www.brokenlinkcheck.com).

^d^Search terms “Erosion”, “Erosionen”, “erosive Zahnhartsubstanzdefekte”, “erosiver Zahnhartsubstanzverlust”, “Säureschädigung”, and “Säureschäden” were tested.
