# Supplementary material for: Quality of Patient-Centered eHealth Information on Erosive Tooth Wear: Systematic Search and Evaluation of Websites and YouTube Videos
Source: J Med Internet Res. 2024 Jan 31;26:e49514. doi: 10.2196/49514 (PMC10867746; doi:10.2196/49514)
Supplement: Multimedia Appendix 3 [file jmir_v26i1e49514_app3.doc]

**Multimedia Appendix 3:** Subdomains regarding the comprehensiveness of information on dental erosion (domain 3) were assessed using a structured checklist. Ordinal scores of 0 (never or no), 1 (sometimes or partially), or 2 (mostly or yes) were given.

| Subdomain | Item | Median (IQR) | Range |
| --- | --- | --- | --- |
| **3.1 Etiology und pathogenesis** | | | |
|  | Are extrinsic factors given? How well are they described? | 1 (0-1) | 0-2 |
|  | Are intrinsic factors given? How well are they described? | 0 (0-1) | 0-2 |
|  | Are specific triggers mentioned (food, products, hobbies)? | 1 (0-1) | 0-2 |
|  | Are further cofactors (eg, reduced saliva flow rate) given? | 0 (0-0) | 0-2 |
| **3.2 Clinical signs** | | | |
|  | Are clinical signs given? Are pictures/schematic drawings shown? | 0 (0-1) | 0-2 |
|  | Are preferred localizations of erosive tooth wear given? | 0 (0-0) | 0-2 |
|  | Is a differentiation to other forms of non-carious tooth defects (ie, abrasion and attrition) made? | 0 (0-0) | 0-2 |
| **3.3 Preventive measures** | | | |
|  | Are preventive measures (eg, regarding food consumption, food additives) given? | 0 (0-0) | 0-2 |
|  | Are adapted consumption modes (eg, rinsing with water, limiting consumption of erosive food or beverages to the main meals) given? | 0 (0-0) | 0-2 |
|  | Are recommendations for personal dental hygiene given? Are specific erosion-protective products recommended? | 0 (0-1) | 0-2 |
| **3.4 Therapy options** | | | |
|  | Is information given? At what severity is it indicated? | 0 (0-0) | 0-1 |
|  | Are non-restorative treatment (ie, preventive) measures given? At what severity are they indicated? | 0 (0-0) | 0-1 |
|  | Are restorative treatment measures mentioned? At what severity are they indicated? | 0 (0-1) | 0-1 |
