# Supplementary material for: Quality of Patient-Centered eHealth Information on Erosive Tooth Wear: Systematic Search and Evaluation of Websites and YouTube Videos
Source: J Med Internet Res. 2024 Jan 31;26:e49514. doi: 10.2196/49514 (PMC10867746; doi:10.2196/49514)
Supplement: Multimedia Appendix 4 [file jmir_v26i1e49514_app4.doc]

**Multimedia Appendix 4:** Subdomains regarding generic quality and risk of bias (domain 4) were assessed using the DISCERN instrument [40]. Ordinal scores of 1 (never or no), 2 (sometimes), 3 (partially), 4 (mostly), or 5 (always or yes) were given.

| Subdomain | Item | Median (IQR) | Range |
| --- | --- | --- | --- |
| **4.1 Reliability** | | | |
|  | Are the aims clear? | 1 (1-2) | 1-5 |
|  | Does it^a^ achieve its aims? | 0 (0-3) | 0-5 |
|  | Is it relevant? | 3 (2-3) | 1-5 |
|  | Is it clear what sources of information were used to compile the publication? | 1 (1-2) | 1-4 |
|  | Is it clear when the information used or reported in the publication was produced? | 1 (1-2) | 1-5 |
|  | Is it^a^ balanced and unbiased? | 2 (2-3) | 1-4 |
|  | Does it^b^ provide details of additional sources of support and information? | 1 (1-2) | 1-4 |
|  | Does it^a^ refer to areas of uncertainty? | 1 (1-1) | 1-3 |
| **4.2 Quality** | | | |
|  | Does it^a^ describe how each treatment works? | 2 (1-3) | 1-4 |
|  | Does it^a^ describe the benefits of each treatment? | 2 (1-3) | 1-5 |
|  | Does it^a^ describe the risks of each treatment? | 1 (1-1) | 1-4 |
|  | Does it^a^ describe what would happen if no treatment is used? | 2 (1-2) | 1-4 |
|  | Does it^a^ describe how the treatment choices affect overall quality of life? | 1 (1-1) | 1-3 |
|  | Is it clear that there may be more than one possible treatment choice? | 1 (1-2) | 1-5 |
|  | Does it^a^ provide support for shared decision-making? | 2 (1-2) | 1-5 |

^a^Websites’ content.
